# Increased Maternal Genome Dosage Bypasses the Requirement of the FIS Polycomb Repressive Complex 2 in Arabidopsis Seed Development

**DOI:** 10.1371/journal.pgen.1003163

**Published:** 2013-01-10

**Authors:** David Kradolfer, Lars Hennig, Claudia Köhler

**Affiliations:** 1 Department of Plant Biology and Forest Genetics, Uppsala BioCenter, Swedish University of Agricultural Sciences and Linnean Center of Plant Biology, Uppsala, Sweden; 2 Department of Biology and Zurich-Basel Plant Science Center, Swiss Federal Institute of Technology, ETH Centre, Zurich, Switzerland; University of Warwick, United Kingdom

## Abstract

Seed development in flowering plants is initiated after a double fertilization event with two sperm cells fertilizing two female gametes, the egg cell and the central cell, leading to the formation of embryo and endosperm, respectively. In most species the endosperm is a polyploid tissue inheriting two maternal genomes and one paternal genome. As a consequence of this particular genomic configuration the endosperm is a dosage sensitive tissue, and changes in the ratio of maternal to paternal contributions strongly impact on endosperm development. The FERTILIZATION INDEPENDENT SEED (FIS) Polycomb Repressive Complex 2 (PRC2) is essential for endosperm development; however, the underlying forces that led to the evolution of the FIS-PRC2 remained unknown. Here, we show that the functional requirement of the FIS-PRC2 can be bypassed by increasing the ratio of maternal to paternal genomes in the endosperm, suggesting that the main functional requirement of the FIS-PRC2 is to balance parental genome contributions and to reduce genetic conflict. We furthermore reveal that the AGAMOUS LIKE (AGL) gene *AGL62* acts as a dosage-sensitive seed size regulator and that reduced expression of *AGL62* might be responsible for reduced size of seeds with increased maternal genome dosage.

## Author Summary

Flowering plants reproduce by forming seeds that contain an embryo surrounded by a nourishing endosperm tissue that, similar to the mammalian placenta, supports embryo growth. Normal endosperm development requires the FERTILIZATION INDEPENDENT SEED (FIS) Polycomb Repressive Complex2 (PRC2). In most flowering plants the endosperm is a polyploid tissue containing two maternal and one paternal genome copies. As a consequence of this particular genomic configuration the endosperm is a dosage sensitive tissue, and changes in the ratio of maternal and paternal genome copies have drastic effects on endosperm development. Here we investigated the consequences of increased maternal genome dosage on endosperm and seed development. We found that increased maternal genome dosage alleviates the need for the FIS-PRC2 in the endosperm. While in *fis* mutant seeds with normal maternal genome dosage the endosperm fails to cellularize and embryos arrest, in *fis* mutant seeds with increased maternal genome dosage the endosperm cellularizes and viable embryos develop. Our study suggests a functional role of the FIS-PRC2 in balancing parental genome dosage in the endosperm. We propose that the FIS-PRC2 evolved to reduce genetic conflict that arose as a consequence of unbalanced genome contributions in the endosperm.

## Introduction

Seed development in flowering plants is initiated by double fertilization of the female gametophyte. Within the female gametophyte there are two distinct gametic cells that have divergent fates after fertilization. The haploid egg cell will give rise to the diploid embryo, while the homodiploid central cell will form the triploid endosperm [1]. The endosperm supports embryo growth by delivering nutrients acquired from the mother plant [2]. As most angiosperms, the endosperm of *Arabidopsis thaliana* follows the nuclear-type of development where an initial syncytial phase of free nuclear divisions without cytokinesis is followed by cellularization [3]. At the eighth mitotic cycle cellularization of the syncytial endosperm is initiated in the micropylar domain around the embryo, coinciding with the early heart stage of embryo development [4], [5]. The timing of endosperm cellularization correlates with final seed size. Precocious endosperm cellularization results in small seeds, while delayed endosperm cellularization causes the formation of enlarged seeds [6], [7]. Timing of endosperm cellularization can be manipulated by interploidy hybridizations, which have opposite effects on endosperm cellularization and seed size dependent on the direction of the increased parental genome contribution. Increased maternal genome contribution (4n×2n, corresponds to maternal excess hybridization) causes precocious endosperm cellularization and the formation of small seeds. Conversely, increased paternal genome dosage (2n×4n, corresponds to paternal excess hybridization) results in delayed or complete failure of endosperm cellularization, causing seed abortion with an accession-dependent frequency [6], [8], [9]. Developmental defects caused by interploidy hybridizations with increased paternal genome contribution are associated with deregulation of genes that are directly or indirectly controlled by the FERTILIZATION INDEPENDENT SEED (FIS) Polycomb Repressive Complex 2 (PRC2), implicating that developmental aberrations in response to interploidy crosses are largely caused by deregulated FIS-PRC2 target genes [9]. PRC2 is a chromatin-modifying complex that ensures mitotically stable repression of specific target genes by applying trimethylation marks at lysine 27 of histone H3 (H3K27me3) [10], [11]. In plants, several PRC2 subunits are encoded by small gene families that form specific complexes with distinct functions during plant development [10]. The FIS-PRC2 is comprised of the subunits MEDEA (MEA), FERTILIZATION INDEPENDENT SEED2 (FIS2), FERTILIZATION INDEPENDENT ENDOSPERM (FIE) and MULTICOPY SUPPRESSOR OF IRA1 (MSI1) [10]. The FIS-PRC2 complex plays a pivotal role in suppressing initiation of endosperm and seed development in the absence of fertilization [12]–[15]. After fertilization, loss of FIS function causes endosperm overproliferation and cellularization failure, ultimately leading to seed abortion [12], [16], [17]. The phenomenon of decreased seed size in response to maternal excess interploidy hybridizations is known since long [6]; however, the underlying molecular mechanism for this phenomenon remains unknown. A recent study revealed increased expression of the FIS-PRC2 subunit *FIS2* in response to maternal excess hybridizations [18], possibly linking increased FIS-PRC2 activity with decreased seed size. Other recent work proposed increased levels of 24-nt small interfering RNAs (p4-siRNAs) to cause decreased seed size by decreasing expression of *AGAMOUS-LIKE* (*AGL*) MADS-box transcription factor encoding genes in the endosperm [19]. In this study we tested the role of FIS-PRC2 as well as p4-siRNAs in mediating maternal excess interploidy effects. Surprisingly, our study revealed that neither changed levels of FIS-PRC2 nor p4-siRNAs are likely to be involved in mediating effects caused by maternal excess interploidy hybridizations. Instead, our results strongly suggest that reduced *AGL* gene expression as a consequence of reduced paternal genome dosage causes decreased seed size and we reveal that AGL62 acts as a dosage sensitive seed size regulator. We furthermore show that FIS-PRC2 function can be bypassed in maternal excess triploid seeds. Loss of FIS-PRC2 causes the formation of enlarged viable triploid seeds containing a cellularized endosperm and a developed embryo, contrasting the strict requirement of FIS-PRC2 function in diploid seeds. Development of viable *fis* triploid seeds is connected with normalized expression of *AGL* genes, suggesting that reduced *AGL* gene expression as a consequence of reduced paternal genome dosage allows bypassing the need of the FIS-PRC2 complex.

## Results

### Increased Maternal Genome Dosage Caused by the *osd1* Mutation Phenocopies Maternal Excess Interploidy Hybridizations

**Figure 1:**
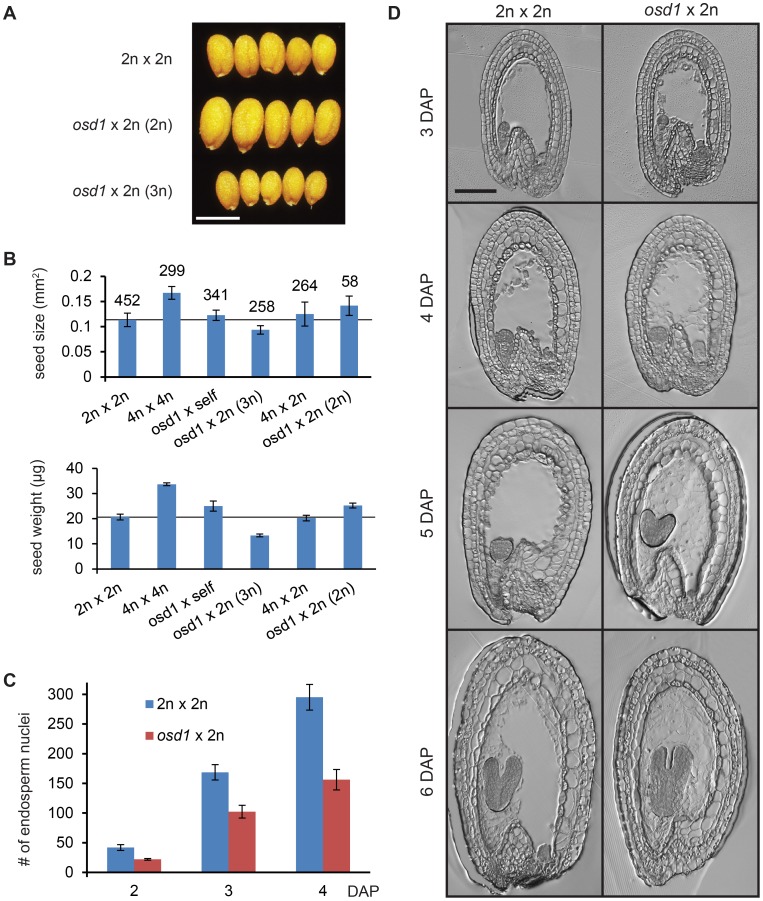
Seeds Derived from Crosses *osd1*×2n Mimic the Effect of Maternal Excess Interploidy Hybridizations. (A) Seeds from crosses of Col 2n×2n (upper panel) and *osd1*×2n (diploid and triploid seeds, middle and lower panel, respectively. Ploidy refers to ploidy of the embryo). Bar = 0.5 mm. (B) Average seed size (upper panel) and seed weight (lower panel) of different crosses. Numbers on top of bars correspond to number of analyzed seeds. For seed weight the average of 100 seeds was calculated in triplicates. Error bars indicate SD. The reference line corresponds to wild-type seed size and weight. (C) Number of endosperm nuclei in Col 2n×2n and *osd1*×2n crosses. n = 5. Error bars indicate SD. (D) Sections of seeds from Col 2n×2n and *osd1*×2n crosses. Bar = 100 µm.

To investigate the effect of increased maternal genome dosage on embryo and endosperm development, we made use of the meiotic *omission of second division* 1 *(osd1)* mutant that forms unreduced diploid male and female gametes at high frequency, whereas the ploidy of the parental plant remains unchanged [20]. Pollinating the *osd1-1* mutant (introgressed into the Col accession) with wild-type pollen allowed us to mimic maternal excess interploidy crosses (4n×2n) without changing the ploidy of the maternal plant. Pollination of an *osd1* plant with wild-type pollen resulted in 91.5% triploid seeds and 8.5% diploid seeds (n = 1921; Table S1), in close agreement with previously published results [20]. Triploid seeds derived from an *osd1*×2n cross were significantly smaller and lighter than diploid wild-type seeds (Figure 1A, 1B, p<0.001). Segregating diploid seeds from the *osd1*×2n cross were significantly bigger than wild-type seeds (p<0.001) (Figure 1A, 1B), maybe because the reduced seed size of the triploid seeds allows diploid sibling seeds to acquire more resources. Alternatively it is possible that loss of OSD1 affects seed size not only by altering ploidy of the endosperm but also by an unknown mechanism in the diploid maternal tissue. Tetraploid seeds derived from self-fertilized *osd1* mutants were only slightly larger than wild-type seeds, contrasting the formation of considerably enlarged seeds by self-fertilized tetraploid plants (Col accession, Figure 1B; p<0.001). This finding suggests that increased size of seeds derived from tetraploid plants is largely caused by maternal sporophytic effects. Triploid seeds derived from 4n×2n interploidy crosses were smaller and lighter compared to tetraploid seeds (p<0.001), but of similar size and weight as wild-type diploid seeds (Figure 1B), contrasting previous data revealing decreased size of maternal excess triploid seeds in Landsberg *erecta* and C24 accession backgrounds [6]. Similar to triploid seeds derived from 4n×2n interploidy crosses [6], triploid seeds derived from *osd1*×2n crosses had a reduced endosperm proliferation rate (Figure 1C) and an early onset of endosperm cellularization (Figure 1D), correlating with decreased seed size compared to the self-fertilized maternal parents. Whereas endosperm cellularization in wild-type diploid and tetraploid seeds started at 6 DAP and was completed at 7–8 DAP, endosperm cellularization in triploid seeds derived from *osd1*×2n as well as 4n×2n crosses started two days earlier at 4 DAP and was completed at 5 DAP (Figure 1D, Figure S1). Precocious endosperm cellularization has previously been connected with small seed size [6], [7], [21], [22], implicating the early onset of endosperm cellularization in triploid seeds as the main cause for reduced seed size. We did not detect reproducible developmental differences of triploid embryos compared to diploid embryos, making it rather unlikely that alteration in triploid embryo development were causally responsible for decreased seed size.

Together, we conclude that increased maternal genome contribution inherited through female gametes is sufficient to cause reduced seed size, establishing the *osd1* mutant as a suitable tool to investigate the effect of maternal excess interploidy hybridizations. While also the *dyad* mutant forms unreduced female gametes and small-sized triploid seeds [23], the very low frequency of viable seed formation (ranging from one to ten viable seeds per *dyad* plant) does not allow detailed investigations of the effect of increased maternal ploidy on seed development using this mutant.

### FIS-PRC2 Target Genes Are Deregulated in Response to Maternal Excess Interploidy Hybridizations

**Figure 2:**
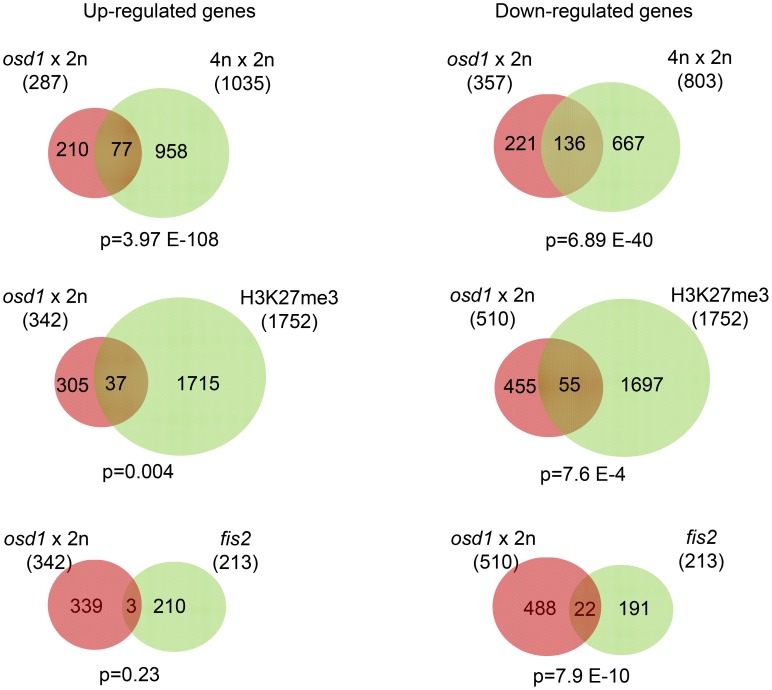
FIS-PRC2 Target Genes Are Deregulated in Response to Maternal Excess Interploidy Hybridizations. Venn diagrams showing overlap of genes being deregulated in seeds derived from *osd1*×2n crosses with genes deregulated in seeds of 4n×2n crosses [24] (upper panel); overlap of deregulated genes in seeds derived from *osd1*×2n crosses with H3K27me3 target genes in the endosperm [49] (middle panel), and genes that are upregulated in *fis2* mutant seeds [9] (lower panel). In the upper panel only deregulated genes that are present on the ATH1 array have been used to calculate the overlap.

Paternal excess interploidy hybridizations cause similar seed developmental defects as mutants lacking FIS-PRC2 function. This is reflected by strikingly similar sets of deregulated genes [9], suggesting failure of FIS-PRC2 function in response to increased paternal genome dosage. As maternal and paternal genome excess cause reciprocal phenotypes [6], we addressed the question whether also maternal excess hybridizations cause global deregulation of FIS-PRC2 target genes. Transcriptome profiling of seeds derived from *osd1*×2n crosses at 6 DAP identified 342 and 510 genes as significantly up- and down-regulated, respectively (Signal Log Ratio (SLR)>1, or SLR<−1, p<0.05; Figure S2, Tables S2 and S3) that significantly overlapped with previously identified genes deregulated in 4n×2n interploidy hybridizations [24] (Table S4). While the overlap of deregulated genes was significant, there was also a high number of non-overlapping genes that are likely a consequence of different tissue types and accession backgrounds used to generate both datasets. Whereas transcriptome data of 4n×2n hybridizations were generated from entire siliques of C24 plants, *osd1*×2n transcriptional profiles were specifically generated from seeds of Columbia plants. Both, up- and down-regulated genes are significantly enriched for the PRC2 hallmark H3K27me3 (Figure 2, Table S2), suggesting a role of FIS-PRC2 in response to maternal excess interploidy hybridizations. We tested whether the genes that are deregulated in interploidy crosses are deregulated also in seeds lacking FIS2 function. Down-regulated genes were significantly enriched for genes affected by loss of FIS2 function, whereas no enrichment was detected for up-regulated genes (Figure 2). Together, the enrichment for H3K27me3 and the overlap with FIS2-responsive genes suggests that the FIS-PRC2 might be involved in mediating the seed phenotype in response to maternal excess hybridizations.

### Decreased Size of Triploid Seeds Persists when FIS-PRC2 Dosage Is Reduced

**Figure 3:**
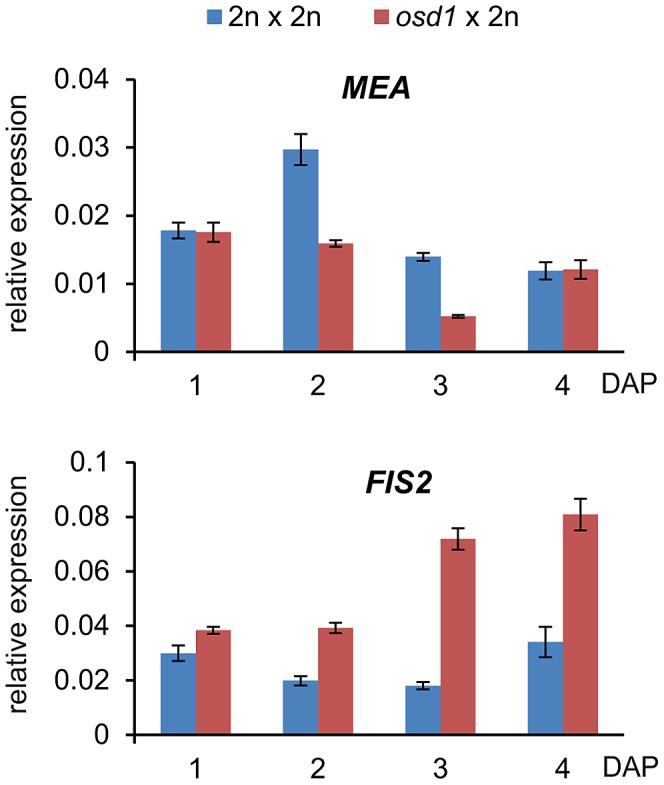
Expression Level of *FIS2* but Not of *MEA* Is Increased in *osd1*×2n Crosses. Quantitative RT-PCR analysis of *MEA* and *FIS2* expression in seeds derived from *osd1*×2n crosses at 1–4 days after pollination (DAP). Error bars indicate s.e.m.

We wished to further test the idea whether decreased expression of FIS2-responsive genes in triploid maternal excess seeds was mediated by increased FIS activity. FIS-PRC2 components FIS2 and MEA are regulated by genomic imprinting and exclusively expressed from the maternally inherited alleles [25]–[27]. Therefore, it was possible that increased maternal genome dosage could cause increased expression of *FIS2* and *MEA* that might in turn be responsible for increased FIS activity. We tested this hypothesis by measuring mRNA levels of *FIS2* and *MEA* in triploid *osd1* seeds and triploid seeds derived from 4n×2n crosses. Levels of *MEA* mRNA were only increased in 4n×2n derived seeds compared to wild-type seeds at 3 DAP, whereas no increase but rather a decrease was detected in triploid *osd1* seeds (Figure 3 and Figure S3). In contrast, *FIS2* mRNA levels were strongly increased in triploid *osd1* seeds and slightly increased in triploid seeds derived from 4n×2n crosses at 2 and 3 DAP (Figure 3 and Figure S3) in agreement with previously published data [18].

**Figure 4:**
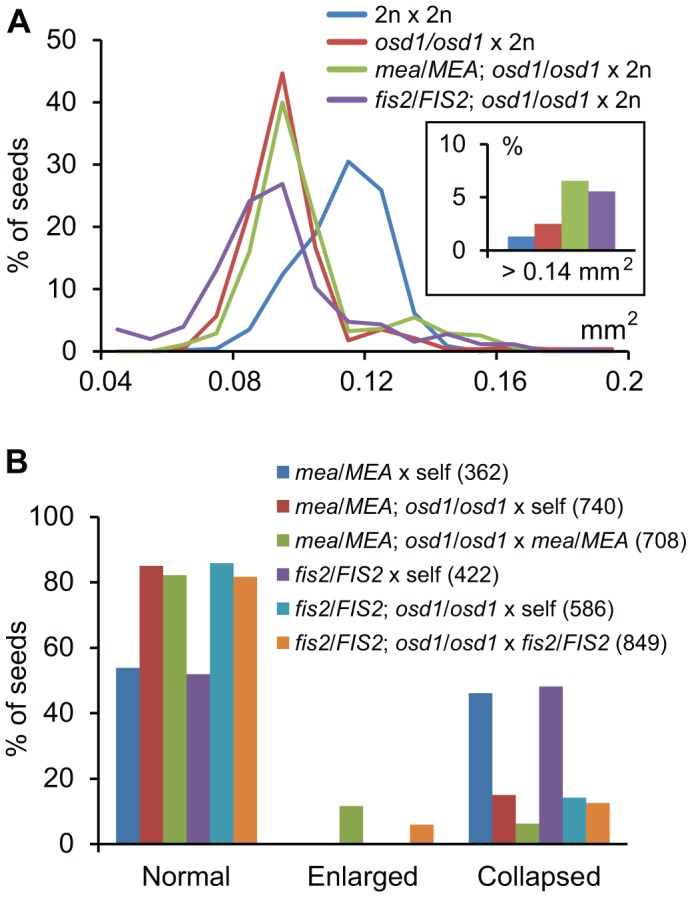
Maternal Excess Triploid *mea* and *fis2* Seeds Are Strongly Enlarged. (A) Distribution of seed sizes from different crosses. A minimum of 250 seeds was analyzed for each cross. Inset shows percentage of seeds larger than 0.14 mm^2^. (B) Percentages of normal, enlarged and collapsed seeds from different crosses. Numbers in parenthesis correspond to numbers of analyzed seeds.

Increased *FIS2* mRNA levels might cause increased FIS-PRC2 activity that in turn could be causally responsible for phenotypic abnormalities of triploid seeds. To test this hypothesis, we generated *mea/MEA; osd1/osd1* and *fis2/FIS2 osd1/osd1* double mutants (Figure S4). *MEA* and *FIS2* are unlinked to the centromere, therefore, mutant and wild-type alleles of both genes will frequently recombine. Consequently, most central cells formed in theses double mutants will be duplex for the *mea* or *fis2* mutation (*mea/mea/MEA/MEA* or *fis2/fis2/FIS2/FIS2*), with a small fraction of central cells being tetraplex for *mea* or *fis2* (*mea/mea/mea/mea* or *fis2/fis2/fis2/fis2*). We pollinated these double mutants with wild-type pollen and analyzed the ploidy and genotype of the resulting progeny. Based on this analysis we infer that about 8% of central cells are nulliplex for *MEA* (tetraplex for *mea*), whereas 10% are nulliplex for *FIS2* (tetraplex for *fis2*) (Table S5). If increased FIS activity was causally responsible for reduced triploid seed size, we expected that reducing the copy number of active *MEA* or *FIS2* alleles should result in the formation of enlarged triploid seeds. More than 70% of seeds derived from pollination of *mea/MEA; osd1/osd1* and *fis2/FIS2; osd1/osd1* with wild-type pollen had only two active maternally inherited wild-type *MEA* or *FIS2* alleles in the pentaploid endosperm, respectively (*mea^M^/mea^M^/MEA^M^/MEA^M^/MEA^P^; fis2^M^/fis2^M^/FIS2^M^/FIS2^M^/FIS2^P^* superscribed M and P correspond to maternal and paternal alleles, Table S5). These seeds were viable and the seed size distribution of the majority of seeds was almost identical to triploid *osd1* seeds (Figure 4A and Figure S5). Based on these results we conclude that increased expression of *FIS2* is unlikely to cause decreased size of triploid seeds.

### Maternal Genome Excess Rescues Lethality of *fis* Mutant Seeds

**Figure 5:**
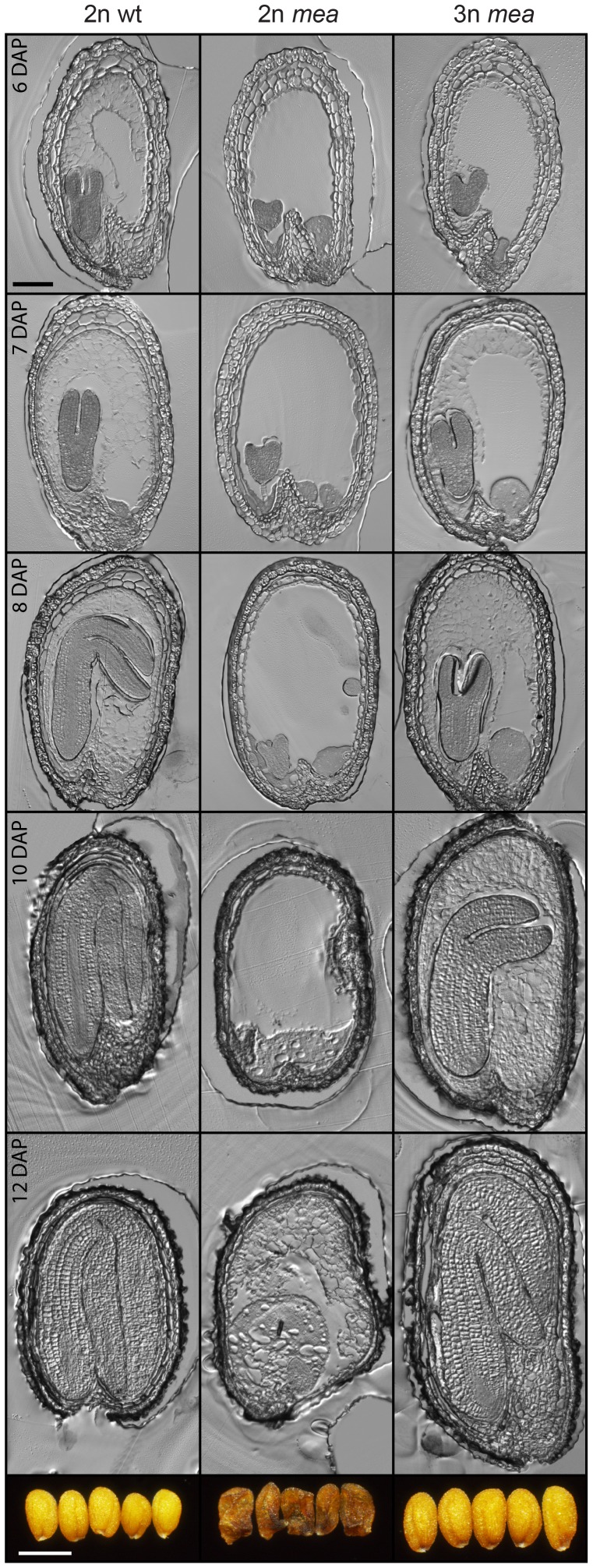
Endosperm Cellularization Is Restored in Triploid *mea* Seeds. Sections of seeds from 2n×2n (left panels), *mea*/*MEA*×2n (middle panels) and *mea*/*MEA*; *osd1*/*osd1*×2n (right panels) crosses at 6–12 days after pollination (DAP). Bar = 100 µm. Images of mature seeds are shown in bottom panels. Bar = 0.5 mm.

We noted that a small fraction of seeds derived from pollination of *mea/MEA; osd1/osd1* and *fis2/FIS2; osd1/osd1* with wild-type pollen were strongly enlarged compared to triploid *osd1* seeds (Figure 4A, Figure 5, Figure S6). Similarly, we detected about 10% and 5% of enlarged seeds in the progeny of the cross *mea/MEA; osd1/osd1*×*mea/MEA* and *fis2/FIS2; osd1/osd1*×*fis2/FIS2*, respectively (Figure 4B) that were not detected in the progeny of self-fertilized *mea/MEA* and *fis2/FIS2* mutants (Figure 4B). About 4.3% of seeds derived from crosses *mea/MEA; osd1/osd1*×*mea/MEA* are expected to be diploid *mea* seeds (Tables S1 and S5), corresponding closely to the observed 5% of collapsed seeds that largely failed to germinate (only 1 out of 35 tested collapsed seeds germinated). We analyzed ploidy and genotype of enlarged seeds derived from crosses *mea/MEA; osd1/osd1*×*mea/MEA*. Genotyping revealed them being either duplex or triplex for the *mea* mutation (*mea^M^/mea^M^/MEA^P^*; *mea^M^/mea^M^/mea^P^*; Figure S7), revealing that triploid seeds can bypass the requirement of MEA function. Among collapsed *fis2* seeds we found that 25% (n = 76) of those seeds were able to germinate. Similar to enlarged *mea* seeds, ploidy analysis and genotyping revealed that these seeds were triploid seeds duplex or triplex for the *fis2* mutation (*fis2^M^/fis2^M^/FIS2^P^; fis2^M^/fis2^M^/fis2^P^*; Figure 4B, Figure S7), adding strong support to the view that triploid seeds can bypass the requirement of FIS-PRC2 function. In contrast, self-fertilized *mea/MEA; osd1/osd1* and *fis2/FIS2; osd1/osd1* mutants did not form enlarged seeds and none of the collapsed seeds was able to germinate, indicating that bypass of FIS function depends on an increased ratio of maternal to paternal genomes, rather than an absolute increase of maternal genome dosage.

Lethality of *fis* mutant seeds is associated with a failure of endosperm cellularization [28], [29]. We asked the question whether rescue of *mea* and *fis2* mutant triploid seeds would be associated with a restoration of endosperm cellularization. Indeed, we found that in contrast to diploid *mea* and *fis2* seeds, endosperm cellularization of triploid duplex or triplex *mea* and *fis*2 seeds was initiated and completed, albeit it occurred delayed compared to wild-type seeds. Whereas cellularization of wild-type seeds was largely progressed at 6 DAP, it was only initiated in triploid *mea* and *fis2* seeds at this time and was completed only at 10 DAP (Figure 5 and Figure S6).

Together, we conclude that relative increase of maternal to paternal genome dosage allows bypass of FIS function and restores endosperm cellularization in *fis* mutant seeds.

### Expression of *AGL* MADS Box Genes Is Normalized in Triploid *mea* and *fis2* Seeds

**Figure 6:**
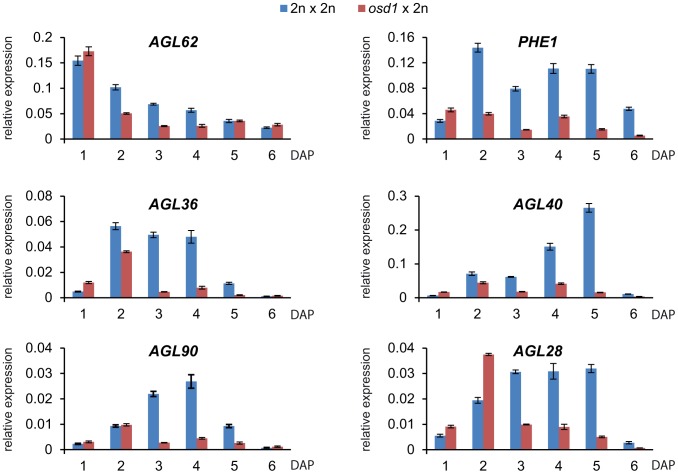
Expression of *AGL* MADS Box Genes Is Decreased in *osd1*×2n Crosses. Quantitative RT-PCR analysis of *AGL62*, *PHE1*, *AGL90*, *AGL36*, *AGL40* and *AGL28*. Error bars indicate s.e.m.

Endosperm cellularization is negatively regulated by the MADS-box transcription factor AGL62, and complete loss of AGL62 causes precocious endosperm cellularization after few mitotic divisions [30]. We addressed the question whether precocious endosperm cellularization in triploid seeds correlates with decreased expression levels of *AGL62*. In agreement with this notion, expression of *AGL62* was reduced in triploid *osd1* seeds at timepoints before cellularization at 5 DAP (Figure 6), similar to decreased expression of *AGL62* in 4n×2n interploidy hybridizations [19] (Figure S8). Yeast two-hybrid interaction studies revealed that AGL62 interacts directly with AGL transcription factors such as the PEG PHERES1 (PHE1), the MEG AGL36, and AGL90. AGL62 also interacts indirectly with the MEG AGL28 and AGL40 that both directly interact with PHE1 [31], [32]. We tested whether expression of the genes encoding direct and indirect interaction partners of AGL62 was altered in triploid seeds. Similar to the reduced expression of *AGL62*, expression of all tested *AGL* genes was strongly reduced in triploid *osd1* seeds as well as triploid seeds derived from 4n×2n crosses (Figure 6 and Figure S8).

**Figure 7:**
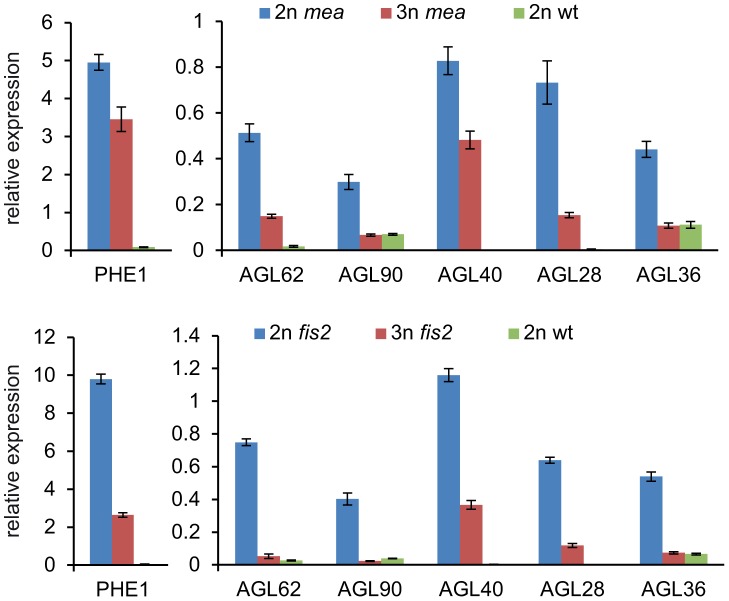
Expression of *AGL* MADS Box Genes Is Normalized in Triploid *mea* and *fis2* Seeds. Quantitative RT-PCR analysis of *AGL62*, *PHE1*, *AGL90*, *AGL40*, *AGL28* and *AGL36* at 8 DAP in *mea* and *fis2* seeds (upper and lower panel, respectively). Enlarged seeds with a *fis* phenotype were selected as 3n *mea* and *fis2* seeds from *mea*/*MEA*; *osd1*/*osd1*×2n and *fis2*/*FIS2*; *osd1*/*osd1*×2n crosses, respectively. Diploid *mea* seeds are a mixture of wild-type and *mea* seeds. Error bars indicate s.e.m.

Reduced maternal genome dosage of *AGL62* can suppress *fis2* seed abortion [29] likely by initiating endosperm cellularization. We therefore addressed the question whether restoration of endosperm cellularization in triploid *mea* and *fis2* seeds is accompanied by normalized expression of *AGL* genes. Because of the extreme size difference, triploid and diploid *mea* and *fis2* seeds can easily be distinguished from other seeds at 8 DAP and manually isolated. Expression of six *AGL* genes implicated in endosperm cellularization was measured, and all tested genes had reduced transcript levels in triploid *mea* and *fis2* seeds (Figure 7), correlating with the initiation of endosperm cellularization (Figure 5). Together, we conclude that decreased *AGL* gene expression in triploid maternal excess seeds alleviates the need for FIS-PRC2 function.

### Reduced Size of Triploid Seeds Is Not Dependent on the p4-siRNA Pathway

**Figure 8:**
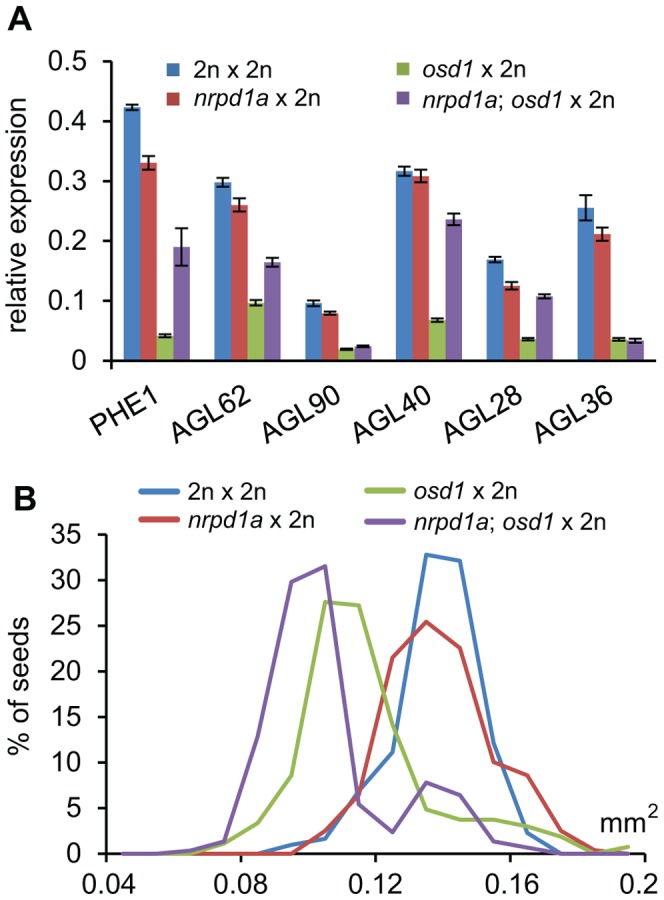
Maternal Loss of NRPD1a Does Not Restore Wild-Type Levels of *AGL* Expression and Wild-Type Seed Size. (A) Quantitative RT-PCR analysis of *PHE1*, *AGL62*, *AGL90*, *AGL40*, *AGL28* and *AGL36* in seeds derived from crosses 2n×2n, *nrpd1a/nrpd1a*×2n, *osd1/osd1*×2n, and *nrpd1a/nrpd1a; osd1/osd1*×2n at 3 DAP. Error bars indicate s.e.m. (B) Distribution of seed sizes from different crosses. A minimum of 250 seeds was analyzed for each cross.

Previous work proposed a possible connection of increased levels of p4-siRNAs small interfering RNAs (siRNAs) in seeds derived from 4n×2n hybridizations and decreased expression levels of *AGL* genes [19]. Biosynthesis of p4-siRNAs is dependent on RNA polymerase IV (PolIV) encoded by *NRPD1a* [33], [34]. To test the requirement of p4- siRNAs for dampening expression of *AGL* genes, we generated *nrpd1a/nrpd1a; osd1/osd1* double mutants and pollinated double homozygous mutants with wild-type pollen. In seeds resulting from this cross at 3 DAP expression of *AGL62*, *PHE1*, *AGL28* and *AGL40* was increased compared to wild-type triploid seeds, but remained significantly below wild-type expression levels (p<0.005; Figure 8A). In contrast, expression levels of *AGL36* and *AGL90* remained unchanged in triploid seeds lacking maternal NRPD1a function (Figure 8A). Maternal loss of NRPD1a in diploid seeds caused decreased expression levels of all tested *AGL* genes except for *AGL40*, which remained expressed at wild-type levels (Figure 8A). We also analyzed the size of seeds derived from *osd1 nrpd1a*×wild-type hybridizations and found that loss of NRPD1 was not connected with increased size of triploid maternal excess seeds (Figure 8B). Together, our results reveal that maternal loss of NRPD1a affects expression of only a subset of *AGL* genes and has no impact on seed size, strongly arguing against a causal role of p4-siRNAs in regulating triploid seed size.

### Reduced Dosage of *AGL62* Causes Reduced Seed Size

**Figure 9:**
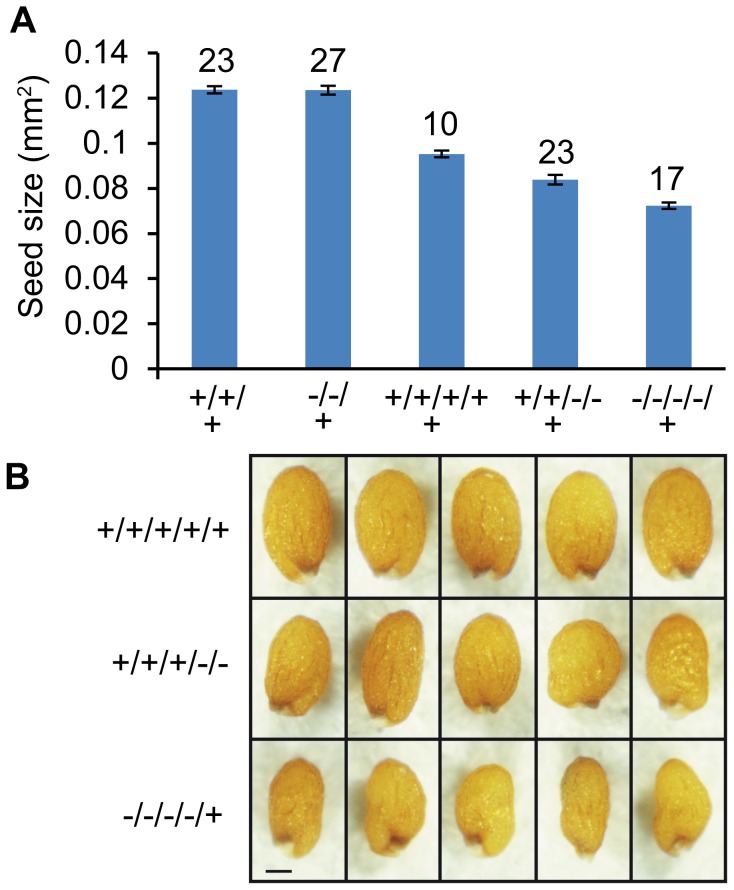
Size of Triploid Seeds Is Dependent on the Dosage of *AGL62*. (A) Size of seeds derived from crosses *agl62*/*AGL62*; *osd1*/*osd1*×wild type and diploid seeds from crosses *agl62*/*AGL62*×wild type. Average seed size of different genotypes is shown. Wild-type (+) and mutant (−) allele frequencies of *AGL62* in the endosperm are shown below the x axis. Numbers on top of bars refer to numbers of analyzed seeds. Error bars indicate s.e.m. (B) Images of triploid seeds from the three categories shown in (A). Bar = 0.1 mm.

Finally, we addressed the question whether AGL62 acts as a dosage dependent seed size regulator. Therefore, we generated *agl62/AGL62; osd1/osd1* double mutants and fertilized them with wild-type pollen. The majority of triploid seeds was found to be simplex for the *agl62* mutation and about 17% to be duplex, corresponding to allele frequencies in the pentaploid endosperm of *agl62^M^/agl62^M^/AGL62^M^/AGL62^M^/AGL62^P^* and *agl62^M^/asgl62^M^/agl62^M^/agl62^M^/AGL62^P^* respectively, Table S6). We analyzed the size of triploid seeds being simplex or duplex for the *agl62* mutation and observed an *AGL62* dosage-dependent decrease in size, with seeds having five functional *AGL62* alleles in the endosperm being larger than seeds with only three or one functional *AGL62* allele (Figure 9A, 9B). In contrast, heterozygous diploid *agl62/AGL62* seeds were similar in size to wild-type seeds (Figure 9A), revealing a differential response of diploid and triploid seeds to reduced AGL62 dosage. If reduced dosage of AGL62 is responsible for decreased size of maternal excess triploid seeds, we expected to find an enrichment of MADS-box binding motifs in those genes that are down-regulated in triploid maternal excess seeds. We tested for the presence of CArG-box motifs of the SRE type (CC(A/T)6GG) as well as of the MEF2-type (CTA(A/T)4TAG), as both have been reported to be bound by plant MADS-box proteins [35], [36]. Among down-regulated genes both motifs were significantly enriched, with 54 (10.6%; Table S2) genes containing a MEF-type motif within 1000 bp upstream of the transcriptional start site, which was significantly higher compared to the genome-wide presence of this motif (6.9%, p<0.001). A similar number of genes contain a SRF-type motif in their promoter region (55 genes, 10.7%, Table S2), which is slightly, but significantly higher compared to the genome-wide frequency of 8.1% (p<0.01). Most strikingly, genes containing a MEF2-type motif in their promoter region were predominantly expressed in the chalazal region of the endosperm (17%; Figure S9), consistent with a preferential expression of AGL62 interacting AGLs in this region of the endosperm [37]. Together, we conclude that AGL62 is a dosage sensitive regulator of triploid seed size, strongly suggesting that reduced expression of *AGL62* causes decreased size of triploid maternal excess seeds.

## Discussion

In this study we report the following new discoveries: (1) The functional requirement of the FIS-PRC2 can be bypassed by increasing the ratio of maternal to paternal genomes. (2) Bypass of FIS-PRC2 function is connected with decreased expression of *AGL62* and interacting AGLs. (3) Decreased seed size of maternal excess triploid seeds is neither mediated by increased activity of FIS-PRC2 and nor by increased levels of p4-siRNAs. (4) Decreased size of maternal excess triploid seeds is likely a consequence of decreased expression of paternally expressed genes. (5) AGL62 is a dosage-sensitive seed size regulator.

The FIS-PRC2 complex is essential for viable seed development; however, the underlying forces that lead to the evolution of the FIS-PRC2 remained obscure. Our study demonstrates that maternal excess triploid seeds can bypass FIS-PRC2 function, suggesting that FIS-PRC2 is mainly required to balance expression of maternally and paternally expressed dosage sensitive genes in the endosperm. Consistent with previous work revealing that *fis* mutant seed abortion can be partially suppressed by maternal loss of *AGL62* [29], we show that normalized *fis* triploid seed development is connected with normalized *AGL62* gene expression. Based on yeast-two-hybrid data AGL62 interacts with several AGLs [31], with at least two of them, *PHE1* and *AGL36* are regulated by genomic imprinting [38], [39]. Therefore, altering the parental genome dosage is expected to impair the balance of *AGL* gene expression. This view is supported by the fact that increased paternal genome dosage causes strongly increased expression of *PHE1*, *AGL62*, and *AGL36* [9], [24], [32], whereas increased maternal genome dosage exerts the converse effect [24] and data shown in this study). Type II MADS-box proteins are well known to form multimeric complexes [40] and changing the expression level of individual members of these complexes strongly impacts on plant development [41]–[43]. It can thus be assumed that unbalanced expression changes of AGL genes in the endosperm will similarly impair functional AGL complex formation and strongly affect endosperm development.

Our study furthermore revealed that decreased seed size in maternal excess interploidy seeds is neither connected with increased FIS-PRC2 function, nor with increased p4-siRNA levels. Instead, we propose that reduced expression of paternally expressed genes causes decreased seed size in maternal excess crosses and conversely, that increased expression of paternally expressed genes causes increased seed size in *fis* mutants. This model predicts that decreasing paternal genome dosage should reduce seed size in *fis* mutants and potentially even partially suppress seed abortion. This prediction is confirmed by the data presented in this study. FIS-PRC2 function can also be bypassed in *fis* seeds that form a sexual embryo and an asexual endosperm [44]. These surviving *fis2* seeds with diploid endosperm remain smaller than wild-type seeds. Our results show that another class of surviving *fis2* seeds, triploid maternal excess *fis* mutant seeds with a pentaploid endosperm, is larger than wild-type seeds. These two classes of surviving *fis2* seeds have two major differences that could explain the very different seed size: First, *fis2* seeds with pentaploid endosperm have four, whereas *fis2* seeds with diploid endosperm have only two maternal genomes. Second, *fis2* seeds with pentaploid endosperm but not those with diploid endosperm contain a paternal genome. Because increased maternal genome dosage usually causes decreased rather than increased seed size, the different maternal genome dosage in the two classes of surviving *fis2* seeds is unlikely causing the observed differences in seed size. Instead, we conclude that the presence of a paternal genome is the main determinant for seed size in *fis2* mutant seeds. Consequently, the FIS-PRC2 regulates paternally contributed seed size regulators that cause endosperm abnormalities and seed abortion upon overexpression. Our work revealed that AGL62 is a dosage-dependent seed size regulator, suggesting that either activation or function of AGL62 depends on a paternally contributed factor. AGL62 physically interacts with the paternally expressed PHE1 [31], raising the possibility that decreased expression of *PHE1* and possibly other proteins reduces the number of functional AGL62 complexes.

Recent data implicate a link between increased levels of PolIV-dependent maternal p4-siRNAs and decreased size of maternal excess seeds [19]. It has been suggested that maternal p4-siRNAs target *AGL* genes and that increased siRNA levels in triploid maternal excess seeds causes decreased *AGL* transcript levels [19]. The results shown in this study reveal that maternal loss of NRPD1a in triploid seeds only affects expression of a subset of *AGL* genes with expression of none of the tested *AGL* genes being restored to wild-type levels. Maternal loss of NRPD1a in triploid seeds did neither affect size of triploid seeds. Therefore, the connection between maternal p4-siRNAs and regulation of *AGL* genes in response to interploidy hybridizations requires further investigations.

Many theoretical considerations argue that endosperms with higher levels of maternal ploidy and reduced levels of interparental genomic conflict have adaptive benefits and should be evolutionary favored [45]–[47]. In agreement with that view, transitions from triploid to higher endosperm ploidy occurred frequently by changing the mode of female gametophyte formation [47]. Based on the data presented in this study we propose that the FIS-PRC2 is needed to counteract excessive parental conflict. Therefore, reduced genetic conflict as a consequence of higher levels of maternal ploidy in the endosperm can bypass the need of the FIS-PRC2. We speculate that the FIS-PRC2 will be of less importance in species forming endosperms with higher maternal ploidy.

## Materials and Methods

### Plant Material and Growth Conditions

Plants were grown in a growth chamber at 60% humidity and daily cycles of 16 h light at 21°C and 8 h darkness at 18°C. *Arabidopsis thaliana* mutants *mea-8* [48], *fis2-5* [49], *agl62-2* [30], and *nrpd1a-3* [34] are in the Columbia accession. The *osd1-1* mutant [20] was kindly provided by Raphael Mercier. The mutant was originally identified in the Nossen background and subsequently introgressed into Columbia by repeated backcrossing over five generations. Tetraploid Columbia plants were kindly provided by Ortrun Mittelsten Scheid. For crosses, designated female partners were emasculated, and the pistils hand-pollinated two day after emasculation.

### RNA Extraction and qPCR Analysis

For analysis of crosses in Figure 7, siliques were opened and a minimum of 50 seeds were harvested into RNA later (Sigma, Buchs, Switzerland). For analysis in all other Figures, three siliques were harvested for each timepoint and frozen in liquid nitrogen.

Glass beads (1.25–1.55 mm) were added, and the samples were ground in a Silamat S5 (IvoclarVivadent, Ellwangen, Germany). RNA was extracted using the RNeasy Plant Mini Kit (Qiagen, Hilden, Germany) according to the manufacturer's instructions. Residual DNA was removed using the Qiagen RNase-free DNase Set and cDNA was synthesized using the Fermentas First strand cDNA synthesis kit (Fermentas, Burlington, Canada) according to the manufacturer's instruction.

Quantitative RT-PCR was performed using an iQ5 Real-Time PCR Detection System (BioRad, Hercules, USA) and Maxima SYBR green qPCR master mix (Fermentas, Burlington, Canada) according to the manufacturer's instruction. Quantitative RT-PCR was performed with three replicates using primers as indicated in Table S7, and results were analyzed as described [50]. Expression of *PP2A* and *ACTIN11* did not change in triploid versus diploid seeds (data not shown), and both genes were used as reference genes with similar results (expression normalized to *PP2A* is shown).

For quantification of *agl62* alleles in triploid seeds, DNA was isolated from seedlings and the number of mutant alleles was determined by quantitative PCR using three different primer pairs (Table S7) for each mutant (one pair that specifically amplifies the T-DNA allele, one pair that amplifies only the wild-type allele and one pair that amplifies both the T-DNA and wild-type allele equally). To distinguish between simplex and duplex mutants, the ratio of T-DNA to wild-type alleles was calculated (Figure S10).

### Microscopy

Tissue sections and clearing analysis were performed as previously described [51]. Pictures were taken using a Leica DMI 4000B microscope and Leica DFC360 FX camera and processed using Adobe Photoshop CS5.

### Seed Size Analysis

Seeds were arranged on glass slides and pictures were taken using a Leica Z16apoA microscope. Images were converted to black and white using the “threshold” function in Adobe Photoshop CS5. Seed size was measured in ImageJ (http://rsbweb.nih.gov/ij/) using the “Analyze Particles” function. Seed size analysis shown in Figure 9A and Figure S5 was done from individual seeds that were later on germinated to determine genotype and ploidy.

### Flow Cytometry

Ploidy levels were measured by flow cytometry with a CyFlow Ploidy Analyzer (Partec, Münster, Germany). For seed ploidy analysis, seeds were allowed to germinate and 10 day old seedlings were analyzed. Plant tissue was chopped with a razor blade in CyStain extraction buffer (Partec), filtered through a 30-µm CellTrics filter (Partec) into a sample tube, and stained with CyStain Staining buffer (Partec).

### Microarray Analysis

#### Samples, array design, and hybridizations

The transcriptional profile of wild-type and *fis2* seeds at 6 DAP has been previously published [9]. Reference transcript profiles during seed development were taken from [52]. To generate transcript profiles of *osd1*×2n crosses and 2n×2n crosses, seeds of at least 20 siliques per sample and three independent biological replicates were harvested into 40 µl RNA*later* (Sigma, Buchs, Switzerland) at 6DAP. RNA extraction and labeling was performed as previously described [9]. AGRONOMICS1 microarrays (Affymetrix, Santa Clara, CA) were hybridized as previously described [53]. Analysis was based upon annotations compiled in TAIR9. Data were deposited into the ArrayExpress database (Accession number E-MTAB-1061).

#### Bioinformatic analysis

All data processing was essentially performed as previously described [9].

Probe sets were called significantly differentially expressed when q<0.05. To enrich for biologically relevant changes, only probe sets with FC_real_>2 or FC_real_<−2 were selected. Data for H3K27me3 target loci were from [49]. The significance of enrichment was estimated based on the hypergeometric test. Analysis of tissue-specificity of differentially expressed genes was performed using MeV4 (http://www.tm4.org/mev/).

## Supporting Information

Figure S1 Seeds Derived from Crosses *osd1*×2n and 4n×2n Cellularize Early. Sections of seeds from Col 2n×2n, *osd1*×2n, Col 4n×2n, and Col 4n×4n crosses. For comparison, images for Col 2n×2n and *osd1*×2n from Figure 1D were included. Bar = 100 µm.
(PDF)

Figure S2 Up- and Down-Regulated Genes in *osd1*×2n Crosses Are Differentially Expressed in the Endosperm. Cluster analysis of genes that were deregulated in seeds derived from *osd1*×2n crosses based on their expression in embryo, endosperm and seed coat during different stages of seed development. Each row represents a gene, and each column represents a tissue type. Tissue types are: embryos from the preglobular(1), globular (2), heart (3), cotyledon (4), and mature stage (5), micropylar (MPE), peripheral (PE) and chalazal (CZE) endosperm derived from seeds containing embryos of the preglobular stage to the mature stage, and seed coat derived from seeds containing embryos of the preglobular stage to the mature stage. Tissue specific expression data are derived from [52]. Red or green indicate tissues in which a particular gene is highly expressed or repressed, respectively. Clustering was done using MeV4 (http://www.tm4.org/mev/).
(PDF)

Figure S3 Expression Levels of *FIS2* and *MEA* Are Not Substantially Changed in 4n×2n Crosses. Quantitative RT-PCR analysis of *MEA* and *FIS2* in seeds derived from 2n×2n, 4n×4, and 4n×2n crosses from 1–4 days after pollination (DAP). Error bars indicate s.e.m.
(PDF)

Figure S4 Scheme of Female Gamete Formation in the *osd1* Mutant. Gametes formed by an *osd1*/*osd1* plant that is heterozygous for another mutation (e.g. *mea*). + and − indicate wild-type and mutant alleles of this mutation. Second meiotic division only occurs in a small fraction of gametes. Recombination is only shown at the position of the respective mutation.
(PDF)

Figure S5 Reducing Maternal *FIS2* Alleles by Half Does Not Alter Triploid Seed Size. Seed size of *fis2*/*FIS2*/*FIS2* (n = 33) and *FIS2/FIS2/FIS2* (n = 23) seeds from a *fis2*/*FIS2*; *osd1*/*osd1*×2n cross. Error bars indicate SD.
(PDF)

Figure S6 Endosperm Cellularization Is Restored in Triploid *fis2* Seeds. Sections of wild-type, 2n *fis2* and 3n *fis2* seeds at 7 DAP. Bar = 100 µm. Bottom panels show images of mature seeds. Bar = 0.5 mm.
(PDF)

Figure S7 Triploid Seeds That Are Homozygous Mutant for *mea* or *fis2* Are Viable. Percentage of seeds derived from *mea*/*MEA*; *osd1*/*osd1*×*mea*/*MEA* and *fis2*/*FIS2*; *osd1*/*osd1*×*fis2*/*FIS2* crosses being homozygous mutant (−/−/−) for *mea* or *fis2*or inheriting a wild-type paternal *MEA* or *FIS2* allele (−/−/+). N = 35 (enlarged *mea*), 18 (enlarged *fis2*) and 16 (collapsed *fis2*).
(PDF)

Figure S8 Expression Level of *AGL* MADS Box Genes Is Decreased in 4n×2n Hybridizations. Quantitative RT-PCR analysis of *AGL62*, *PHE1*, *AGL90*, *AGL36*, *AGL40* and *AGL28* in seeds derived from 2n×2n, 4n×4n, and 4n×2n crosses. Error bars indicate s.e.m.
(PDF)

Figure S9 Genes Containing MEF-Type and SRF-Type MADS-Box Binding Motifs Are Differentially Expressed in the Endosperm. Cluster analysis of genes that were down-regulated in seeds derived from *osd1*×2n crosses and contain either MEF-type or SRF-type MADS-box binding motifs based on their expression in embryo, endosperm and seed coat during different stages of seed development. Each row represents a gene, and each column represents a tissue type. Tissue types are: embryos from the preglobular(1), globular (2), heart (3), cotyledon (4), and mature stage (5), micropylar (MPE), peripheral (PE) and chalazal (CZE) endosperm derived from seeds containing embryos of the preglobular stage to the mature stage, and seed coat derived from seeds containing embryos of the preglobular stage to the mature stage. Tissue specific expression data are derived from [52]. Red or green indicate tissues in which a particular gene is highly expressed or repressed, respectively. Clustering was done using MeV4 (http://www.tm4.org/mev/).
(PDF)

Figure S10 Scheme of *AGL62* Allele Frequency Determination. (A) Primer were designed that bind either unspecifically to genomic DNA of wild-type and mutant alleles or specific to only one of them. (B) Expected frequencies during RT-qPCR of wild-type and mutant alleles. The genotype of the embryo is shown on the x-axis.
(PDF)

Table S1 Seeds produced by *osd1*×2n and *osd1*×self crosses. Ploidy was confirmed for several seeds of each category, except for the 3n seeds from *osd1*×self, which are non-viable.
(DOCX)

Table S2 Genes up- and down-regulated in *osd1*×2n (SLR>1; SLR<−1; p<0.05).
(XLSX)

Table S3 GO analysis of deregulated genes in seeds derived from *osd1*×wild type crosses.
(DOCX)

Table S4 Genes commonly deregulated in 4n×2n crosses (Tiwari et al., 2010) and *osd1*×2n (this study).
(XLSX)

Table S5 Genotype of seeds generated by *mea/MEA; osd1/osd1*×2n and *fis2/FIS2; osd1/osd1*×2n crosses. Ploidy and genotype was confirmed for several seeds of each category. Number of 2n seeds was inferred based on the number of 2n seeds segregating in cross *osd1*×wt (Table S1). Maternally inherited alleles are marked in red.
(DOCX)

Table S6 Genotype of seeds generated by an *agl62/AGL62; osd1/osd1*×2n cross. Ploidy and genotype were confirmed for 59 triploid seeds. The number of 2n seeds was inferred based on the number of 2n seeds segregating in an *osd1*×wt control cross (Table S1). Maternally inherited alleles are marked in red.
(DOCX)

Table S7 Primers used in this study.
(DOCX)
