# Supplementary figures and images for: Increased Maternal Genome Dosage Bypasses the Requirement of the FIS Polycomb Repressive Complex 2 in Arabidopsis Seed Development
Source: PLoS Genet. 2013 Jan 10;9(1):e1003163. doi: 10.1371/journal.pgen.1003163 (PMC3542072; doi:10.1371/journal.pgen.1003163)

Figure S1

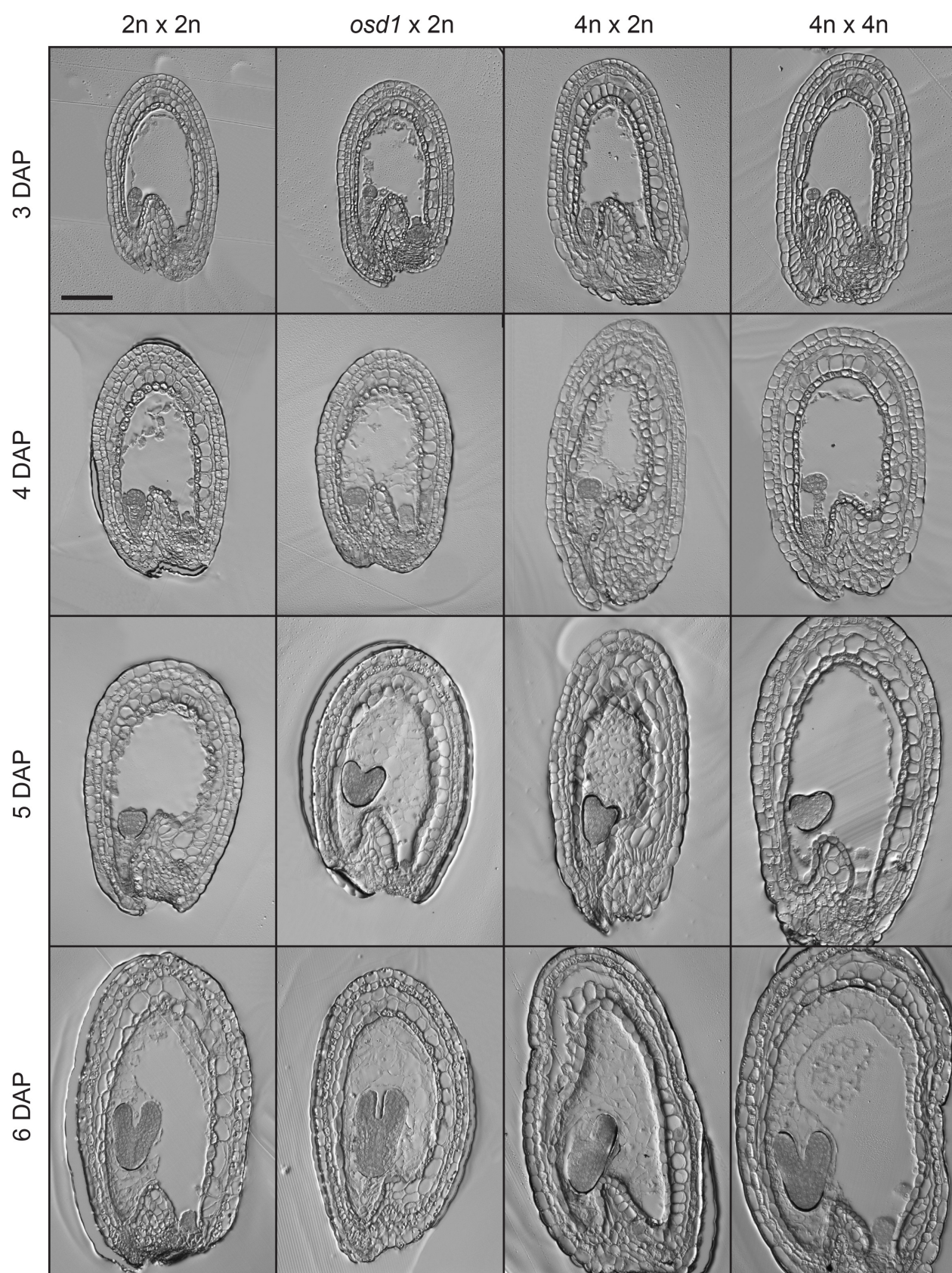

Supplement: Figure S1 — Seeds Derived from Crosses osd1×2n and 4n×2n Cellularize Early. Sections of seeds from Col 2n×2n, osd1×2n, Col 4n×2n, and Col 4n×4n crosses. For comparison, images for Col 2n×2n and osd1×2n from Figure 1D were included. Bar = 100 µm. (PDF) [file pgen.1003163.s001.pdf]

# Figure S3

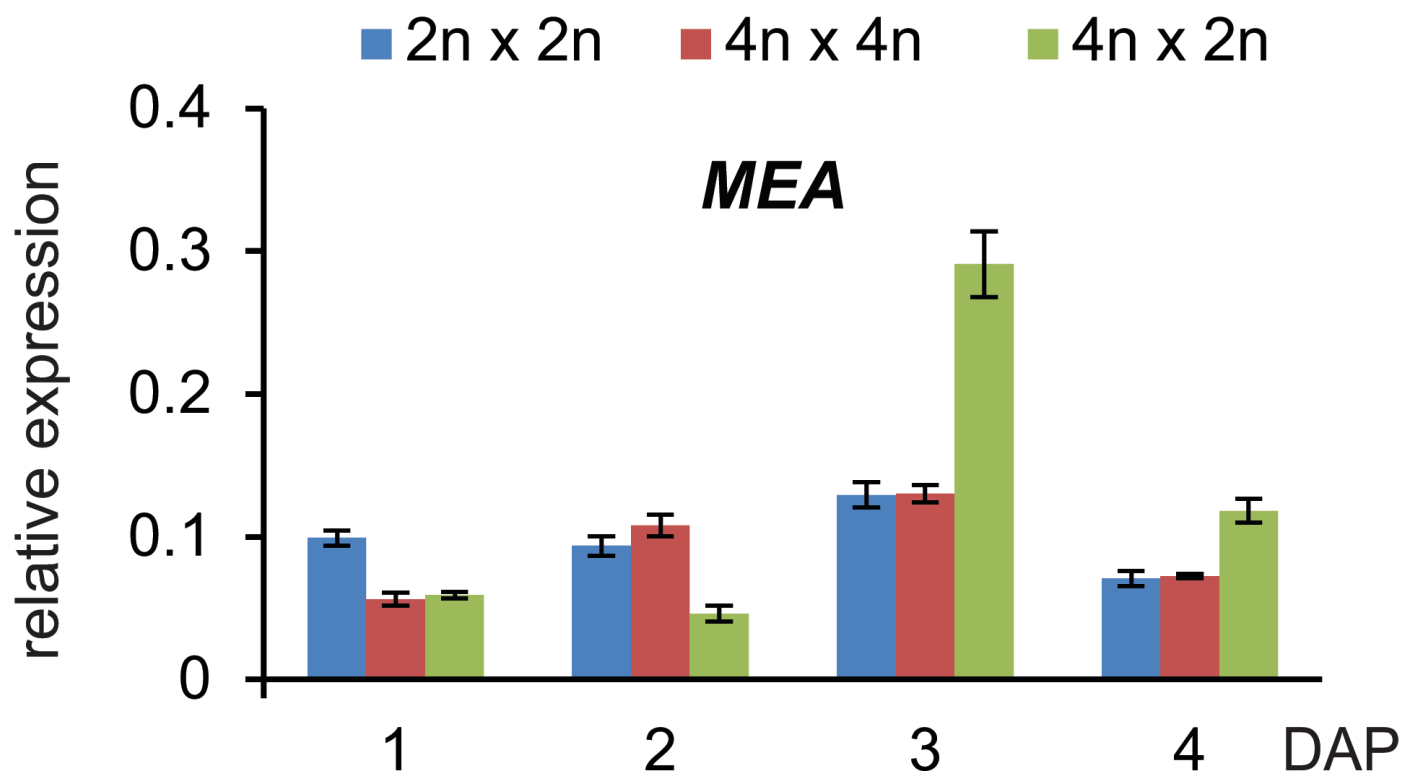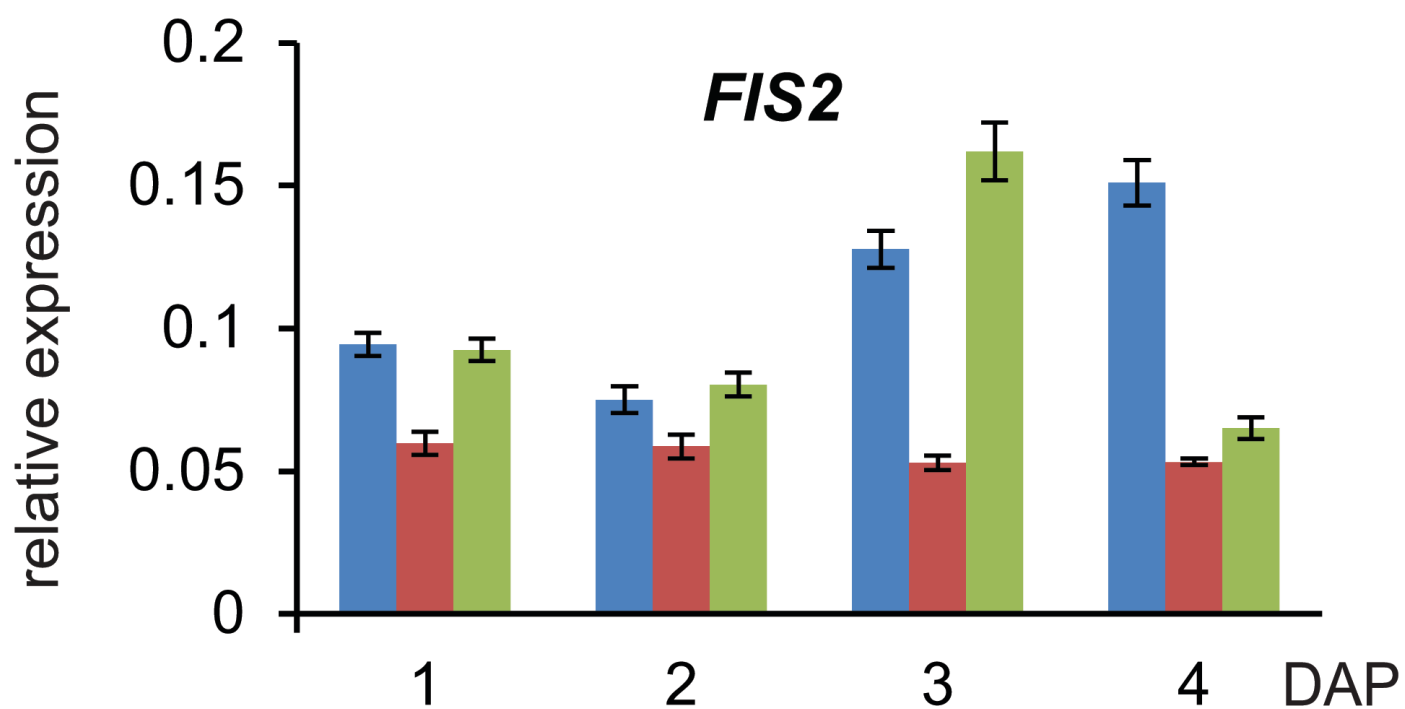

Supplement: Figure S3 — Expression Levels of FIS2 and MEA Are Not Substantially Changed in 4n×2n Crosses. Quantitative RT-PCR analysis of MEA and FIS2 in seeds derived from 2n×2n, 4n×4, and 4n×2n crosses from 1–4 days after pollination (DAP). Error bars indicate s.e.m. (PDF) [file pgen.1003163.s003.pdf]

Figure S4

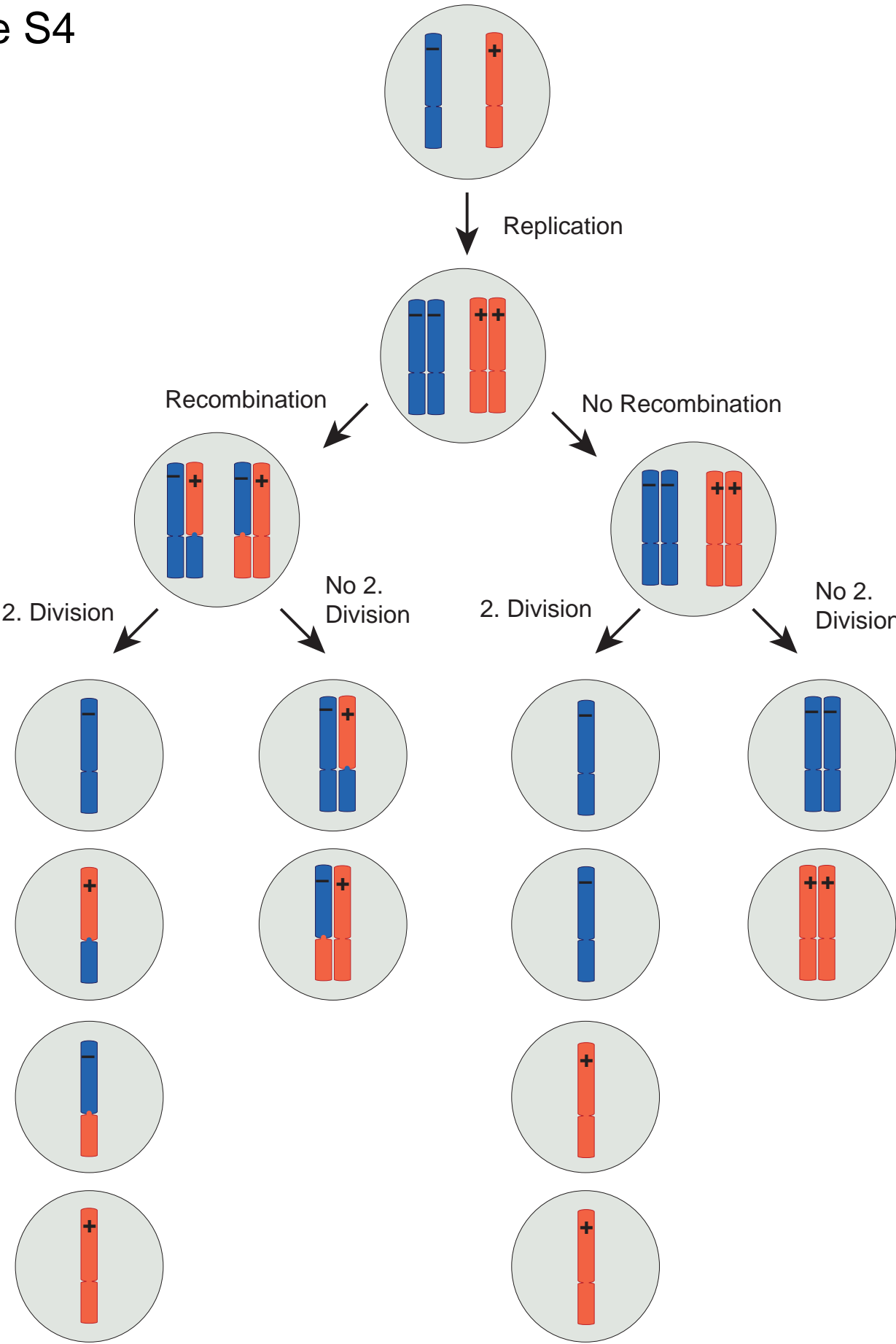

Supplement: Figure S4 — Scheme of Female Gamete Formation in the osd1 Mutant. Gametes formed by an osd1/osd1 plant that is heterozygous for another mutation (e.g. mea). + and − indicate wild-type and mutant alleles of this mutation. Second meiotic division only occurs in a small fraction of gametes. Recombination is only shown at the position of the respective mutation. (PDF) [file pgen.1003163.s004.pdf]

Figure S5

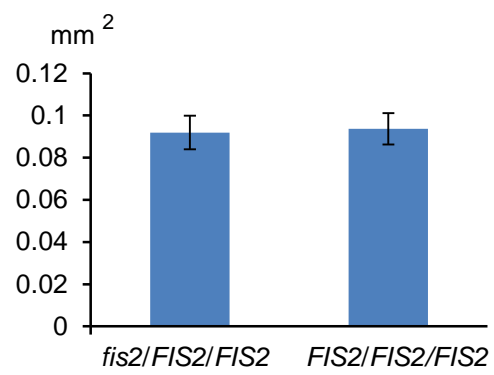

Supplement: Figure S5 — Reducing Maternal FIS2 Alleles by Half Does Not Alter Triploid Seed Size. Seed size of fis2/FIS2/FIS2 (n = 33) and FIS2/FIS2/FIS2 (n = 23) seeds from a fis2/FIS2; osd1/osd1×2n cross. Error bars indicate SD. (PDF) [file pgen.1003163.s005.pdf]

Figure S6

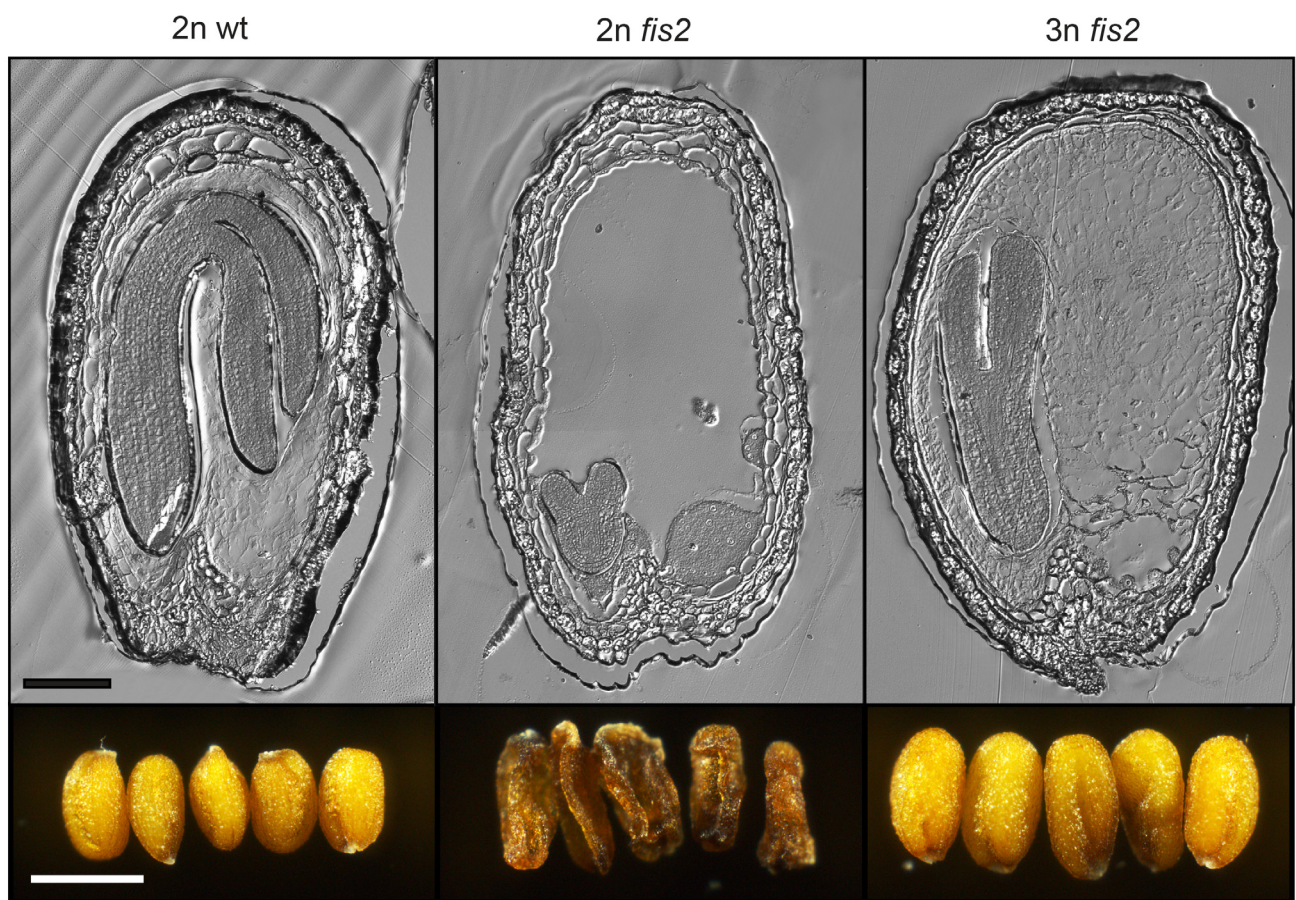

Supplement: Figure S6 — Endosperm Cellularization Is Restored in Triploid fis2 Seeds. Sections of wild-type, 2n fis2 and 3n fis2 seeds at 7 DAP. Bar = 100 µm. Bottom panels show images of mature seeds. Bar = 0.5 mm. (PDF) [file pgen.1003163.s006.pdf]

Figure S7

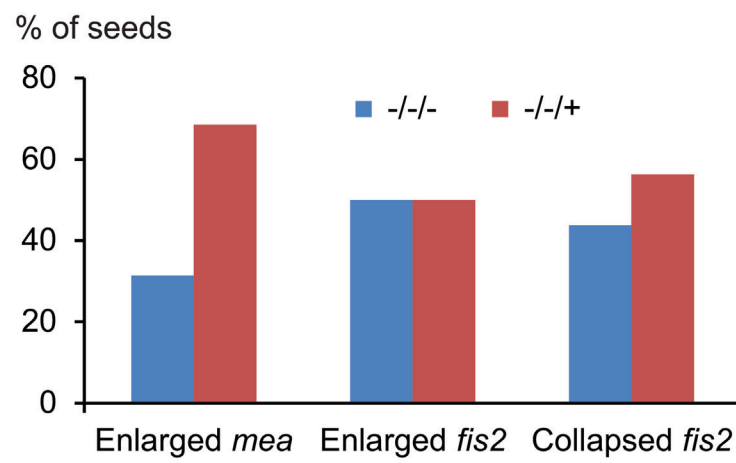

Supplement: Figure S7 — Triploid Seeds That Are Homozygous Mutant for mea or fis2 Are Viable. Percentage of seeds derived from mea/MEA; osd1/osd1×mea/MEA and fis2/FIS2; osd1/osd1×fis2/FIS2 crosses being homozygous mutant (−/−/−) for mea or fis2or inheriting a wild-type paternal MEA or FIS2 allele (−/−/+). N = 35 (enlarged mea), 18 (enlarged fis2) and 16 (collapsed fis2). (PDF) [file pgen.1003163.s007.pdf]

Figure S8

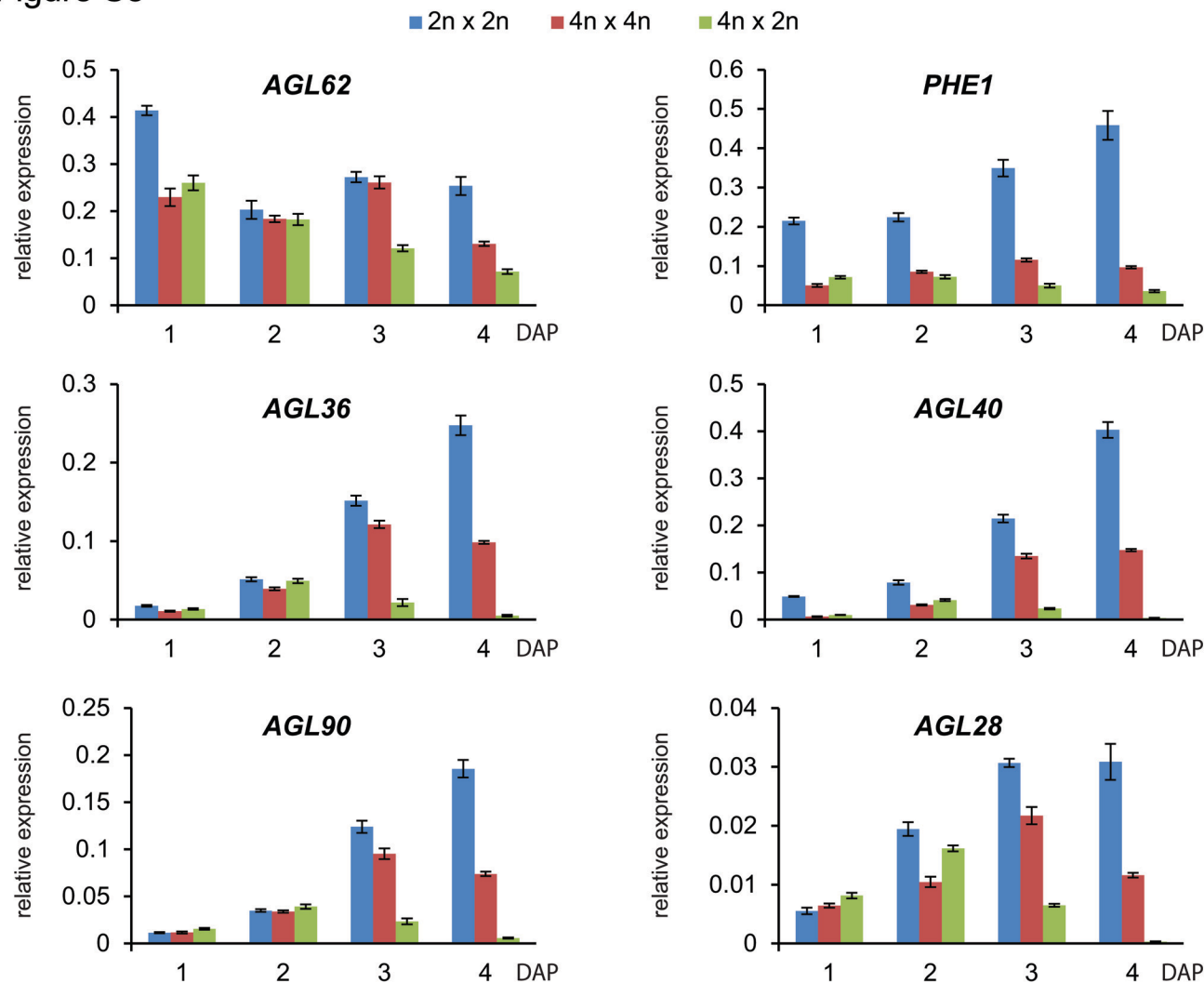

Supplement: Figure S8 — Expression Level of AGL MADS Box Genes Is Decreased in 4n×2n Hybridizations. Quantitative RT-PCR analysis of AGL62, PHE1, AGL90, AGL36, AGL40 and AGL28 in seeds derived from 2n×2n, 4n×4n, and 4n×2n crosses. Error bars indicate s.e.m. (PDF) [file pgen.1003163.s008.pdf]

Figure S9

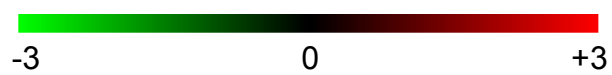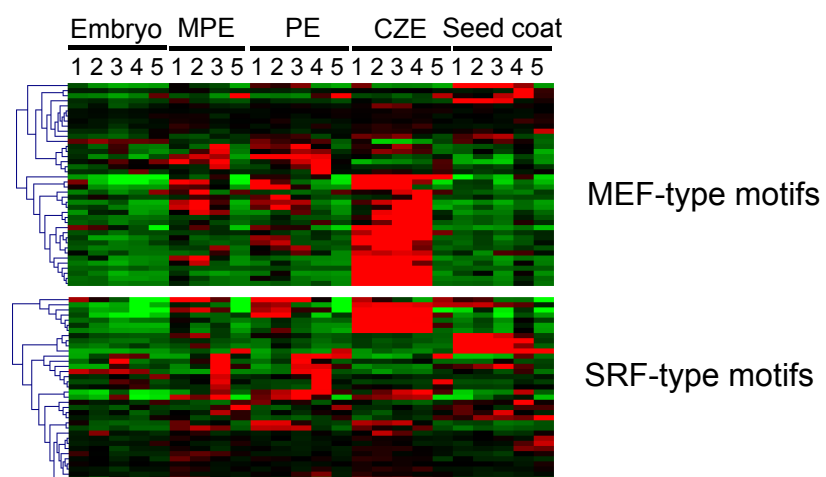

Supplement: Figure S9 — Genes Containing MEF-Type and SRF-Type MADS-Box Binding Motifs Are Differentially Expressed in the Endosperm. Cluster analysis of genes that were down-regulated in seeds derived from osd1×2n crosses and contain either MEF-type or SRF-type MADS-box binding motifs based on their expression in embryo, endosperm and seed coat during different stages of seed development. Each row represents a gene, and each column represents a tissue type. Tissue types are: embryos from the preglobular(1), globular (2), heart (3), cotyledon (4), and mature stage (5), micropylar (MPE), peripheral (PE) and chalazal (CZE) endosperm derived from seeds containing embryos of the preglobular stage to the mature stage, and seed coat derived from seeds containing embryos of the preglobular stage to the mature stage. Tissue specific expression data are derived from [52]. Red or green indicate tissues in which a particular gene is highly expressed or repressed, respectively. Clustering was done using MeV4 (http://www.tm4.org/mev/). (PDF) [file pgen.1003163.s009.pdf]

Figure S10

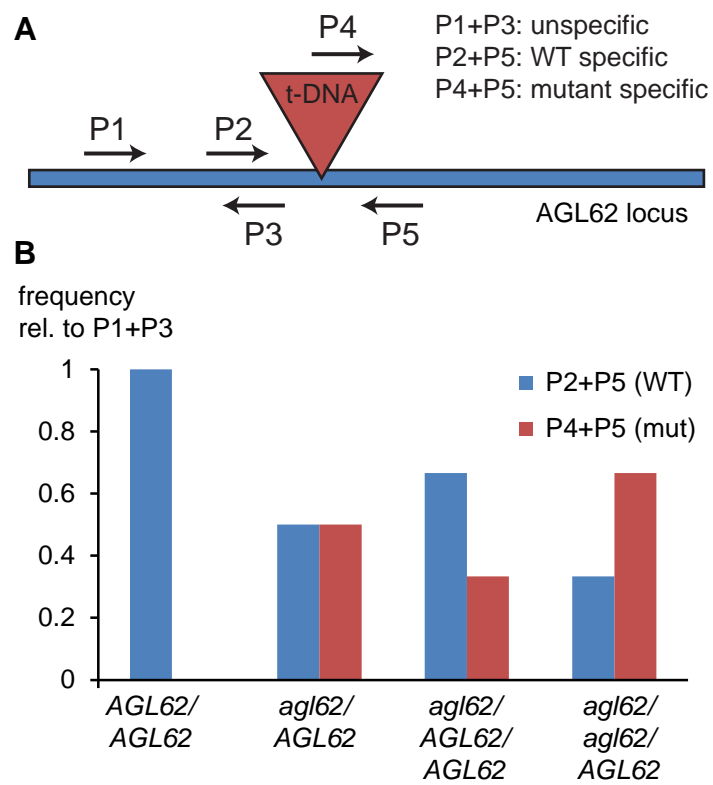

Supplement: Figure S10 — Scheme of AGL62 Allele Frequency Determination. (A) Primer were designed that bind either unspecifically to genomic DNA of wild-type and mutant alleles or specific to only one of them. (B) Expected frequencies during RT-qPCR of wild-type and mutant alleles. The genotype of the embryo is shown on the x-axis. (PDF) [file pgen.1003163.s010.pdf]
