# Supplementary material for: Increased Maternal Genome Dosage Bypasses the Requirement of the FIS Polycomb Repressive Complex 2 in Arabidopsis Seed Development
Source: PLoS Genet. 2013 Jan 10;9(1):e1003163. doi: 10.1371/journal.pgen.1003163 (PMC3542072; doi:10.1371/journal.pgen.1003163)
Supplement: Figure S2 — Up- and Down-Regulated Genes in osd1×2n Crosses Are Differentially Expressed in the Endosperm. Cluster analysis of genes that were deregulated in seeds derived from osd1×2n crosses based on their expression in embryo, endosperm and seed coat during different stages of seed development. Each row represents a gene, and each column represents a tissue type. Tissue types are: embryos from the preglobular(1), globular (2), heart (3), cotyledon (4), and mature stage (5), micropylar (MPE), peripheral (PE) and chalazal (CZE) endosperm derived from seeds containing embryos of the preglobular stage to the mature stage, and seed coat derived from seeds containing embryos of the preglobular stage to the mature stage. Tissue specific expression data are derived from [52]. Red or green indicate tissues in which a particular gene is highly expressed or repressed, respectively. Clustering was done using MeV4 (http://www.tm4.org/mev/). (PDF) [file pgen.1003163.s002.pdf]

Figure S2

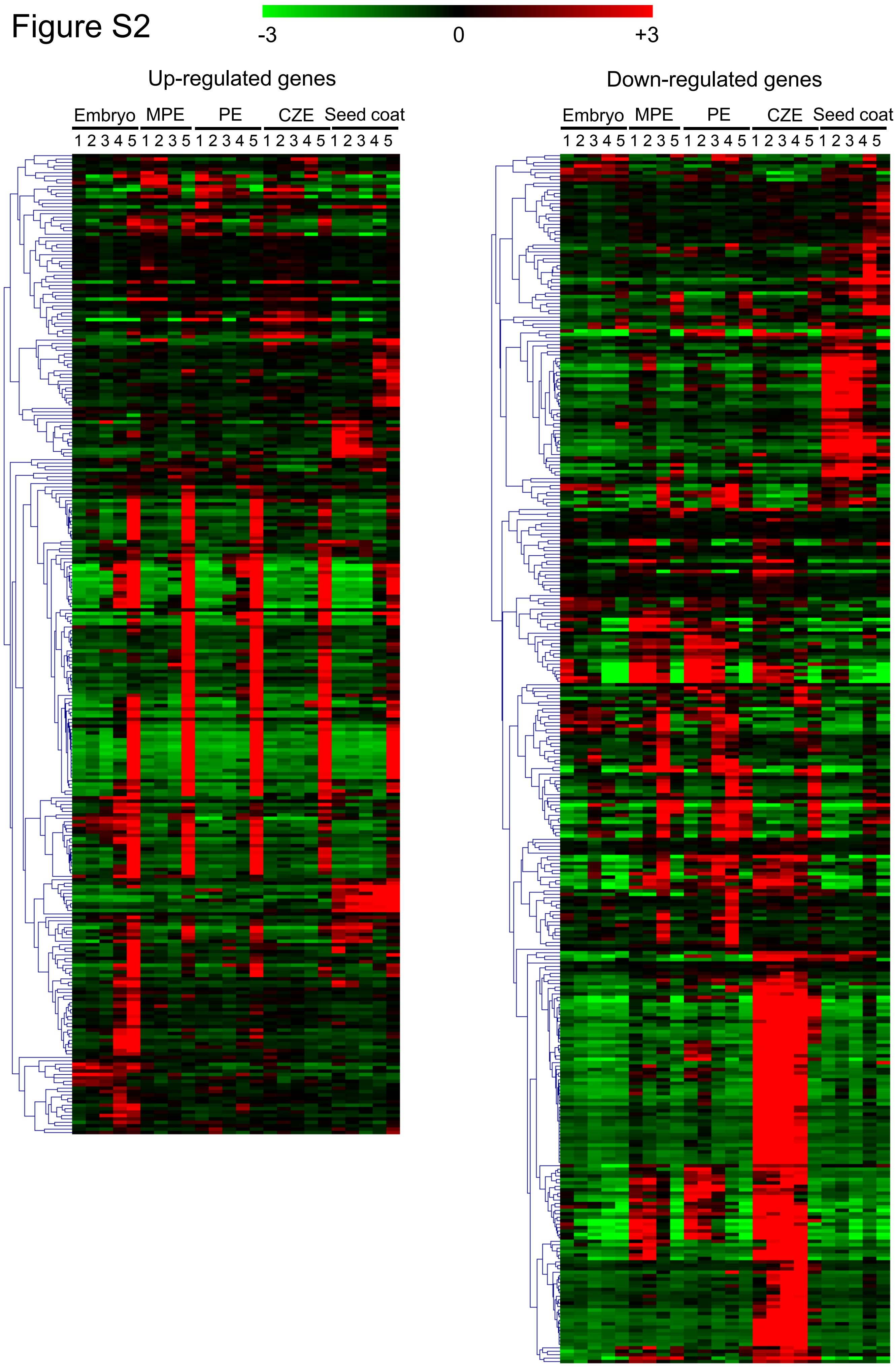
-3
0
+3
Up-regulated genes
Embryo
MPE
PE
CZE
Seed coat
1 2 3 4 5 1 2 3 5 1 2 3 4 5 1 2 3 4 5 1 2 3 4 5
Down-regulated genes
Embryo
MPE
PE
CZE
Seed coat
1 2 3 4 5 1 2 3 5 1 2 3 4 5 1 2 3 4 5 1 2 3 4 5
