# Supplementary material for: Increased Maternal Genome Dosage Bypasses the Requirement of the FIS Polycomb Repressive Complex 2 in Arabidopsis Seed Development
Source: PLoS Genet. 2013 Jan 10;9(1):e1003163. doi: 10.1371/journal.pgen.1003163 (PMC3542072; doi:10.1371/journal.pgen.1003163)
Supplement: Table S1 — Seeds produced by osd1×2n and osd1×self crosses. Ploidy was confirmed for several seeds of each category, except for the 3n seeds from osd1×self, which are non-viable. (DOCX) [file pgen.1003163.s011.docx]

**Table S1**: Seeds produced by *osd1* x 2n and *osd1* x self crosses. Ploidy was confirmed for several seeds of each category, except for the 3n seeds from *osd1* x self, which are non-viable.

|  | Ploidy | Frequency |
| --- | --- | --- |
| *osd1* x 2n | 3n | 91.5% |
| (n=1921) | 2n | 8.5% |
| *osd1* x self | 4n | 91.9% |
| (n=1186) | 3n | 8.1% |
