# Supplementary material for: Increased Maternal Genome Dosage Bypasses the Requirement of the FIS Polycomb Repressive Complex 2 in Arabidopsis Seed Development
Source: PLoS Genet. 2013 Jan 10;9(1):e1003163. doi: 10.1371/journal.pgen.1003163 (PMC3542072; doi:10.1371/journal.pgen.1003163)
Supplement: Table S3 — GO analysis of deregulated genes in seeds derived from osd1×wild type crosses. (DOCX) [file pgen.1003163.s013.docx]

**Table S3. GO analysis of deregulated genes in seeds derived from *osd1* x wild type crosses.**

**Down-regulated genes**

**Biological Process**

| **GO term** | **p-value** | **Number** | **Definition** |
| --- | --- | --- | --- |
| GO:0009407 | 1.69E-06 | 8 | Toxin catabolism |
| GO:0009636 | 1.91E-05 | 8 | Response to toxin |
| GO:0006869 | 1.36E-04 | 9 | Lipid transport |
| GO:0019748 | 6.90E-04 | 16 | Secondary metabolism |
|  |  |  |  |

**Molecular function**

| GO:0004364 | 2.78E-06 | 8 | Glutathione transferase activity |
| --- | --- | --- | --- |
| GO:0008289 | 2.85E-06 | 13 | Lipid binding |
| GO:0005543 | 4.15E-04 | 5 | Phospholipid binding |

**Up-regulated genes**

**Biological process**

| **GO term** | **p-value** | **Number** | **Definition** |
| --- | --- | --- | --- |
| GO:0045735 | 8.71E-14 | 14 | Nutrient reservoir activity |
| GO:0008289 | 3.57E-08 | 13 | Lipid binding |
| GO:0016491 | 3.50E-04 | 30 | Oxidoreductase activity |
| GO:0005351 | 5.61E-03 | 5 | Sugar porter activity |
| **Molecular function** | | | |
| GO:0006869 | 8.21E-08 | 11 | Lipid transport |
| GO:0009664 | 4.11E-04 | 7 | Cell wall organization and biogenesis |
| GO:0019748 | 1.38E-03 | 12 | Secondary metabolism |
| GO:0006629 | 4.48E-03 | 16 | Lipid metabolism |
| GO:0008299 | 5.61E-03 | 5 | Isoprenoid biosynthesis |
