# Supplementary material for: Increased Maternal Genome Dosage Bypasses the Requirement of the FIS Polycomb Repressive Complex 2 in Arabidopsis Seed Development
Source: PLoS Genet. 2013 Jan 10;9(1):e1003163. doi: 10.1371/journal.pgen.1003163 (PMC3542072; doi:10.1371/journal.pgen.1003163)
Supplement: Table S5 — Genotype of seeds generated by mea/MEA; osd1/osd1×2n and fis2/FIS2; osd1/osd1×2n crosses. Ploidy and genotype was confirmed for several seeds of each category. Number of 2n seeds was inferred based on the number of 2n seeds segregating in cross osd1×wt (Table S1). Maternally inherited alleles are marked in red. (DOCX) [file pgen.1003163.s015.docx]

**Table S5**: Genotype of seeds generated by *mea*/*MEA*; *osd1/osd1* x 2n and *fis2*/*FIS2*; *osd1/osd1* x 2n crosses. Ploidy and genotype was confirmed for several seeds of each category. Number of 2n seeds was inferred based on the number of 2n seeds segregating in cross *osd1 x wt* (Table S1). Maternally inherited alleles are marked in red.

|  | F1 Genotype | Endosperm genotype | Ploidy | Frequency | |
| --- | --- | --- | --- | --- | --- |
| *mea*/*MEA*; *osd1/osd1* x 2n | *MEA/MEA/MEA* | *MEA/MEA/MEA/MEA/MEA* | 3n | | 8.3% |
| (n=100) | *mea/mea/MEA* | *mea/mea/MEA/MEA/MEA* | 3n | | 74.9% |
|  | *mea/mea/MEA* | *mea/mea/mea/mea/MEA* | 3n | | 8.3% |
|  | *MEA/MEA* or *mea/MEA* | *MEA/MEA/MEA or mea/mea/MEA* | 2n | | 8.5% |
| *fis2*/*fis2*; *osd1/osd1* x 2n | *FIS2/+/+* | *FIS2/FIS2/FIS2/FIS2/FIS2* | 3n | | 10% |
| (n=147) | *fis2/fis2/FIS2* | *fis2/fis2/FIS2/FIS2/FIS2* | 3n | | 71.5% |
|  | *fis2/fis2/FIS2* | *fis2/fis2/fis2/fis2/FIS2* | 3n | | 10% |
|  | *FIS2/FIS2* or *fis2/fis2* | *FIS2/FIS2/FIS2 or fis2/fis2/FIS2* | 2n | | 8.5% |
