# Supplementary material for: Increased Maternal Genome Dosage Bypasses the Requirement of the FIS Polycomb Repressive Complex 2 in Arabidopsis Seed Development
Source: PLoS Genet. 2013 Jan 10;9(1):e1003163. doi: 10.1371/journal.pgen.1003163 (PMC3542072; doi:10.1371/journal.pgen.1003163)
Supplement: Table S6 — Genotype of seeds generated by an agl62/AGL62; osd1/osd1×2n cross. Ploidy and genotype were confirmed for 59 triploid seeds. The number of 2n seeds was inferred based on the number of 2n seeds segregating in an osd1×wt control cross (Table S1). Maternally inherited alleles are marked in red. (DOCX) [file pgen.1003163.s016.docx]

**Table S6:**   Genotype of seeds generated by an *agl62*/*AGL62*; *osd1/osd1* x 2n cross. Ploidy and genotype were confirmed for 59 triploid seeds. The number of 2n seeds was inferred based on the number of 2n seeds segregating in an *osd1 x wt* control cross (Table S1). Maternally inherited alleles are marked in red.

|  | F1 Genotype | Endosperm genotype | Ploidy | Frequency |
| --- | --- | --- | --- | --- |
| *agl62*/*AGL62*; *osd1/osd1* x 2n | *AGL62/ AGL62/AGL62* | *AGL62/AGL62/AGL62/AGL62/AGL62* | 3n | 16% |
| (n=82) | *agl62/agl62/AGL62* | *agl62/agl62/AGL62/AGL62/AGL62* | 3n | 60% |
|  | *agl62/agl62/AGL62* | *agl62/agl62/agl62/agl62/AGL62* | 3n | 16% |
|  | *AGL62/AGL62* or *agl62/AGL62* | *AGL62/AGL62/AGL62 or agl62/agl62/AGL62* | 2n | 8% |
