# Supplementary material for: Increased Maternal Genome Dosage Bypasses the Requirement of the FIS Polycomb Repressive Complex 2 in Arabidopsis Seed Development
Source: PLoS Genet. 2013 Jan 10;9(1):e1003163. doi: 10.1371/journal.pgen.1003163 (PMC3542072; doi:10.1371/journal.pgen.1003163)
Supplement: Table S7 — Primers used in this study. (DOCX) [file pgen.1003163.s017.docx]

**Table S7**. Primers used in this study.

Primers for expression analysis

| *AGL62* | CCTCCTCACCAACACAACAA |
| --- | --- |
|  | ACCTTTGAACCCCTCGAGTT |
| *PHE1* | TCCAACACCGAAAACTCCAT |
|  | TCCAACACCGAAAACTCCAT |
| *AGL36* | GTGCTCTCATCTACAGTCCA |
|  | CATCATCTTCTTGGTTCGGG |
| *AGL90* | CTTGTGCTCTCATCTATAGTCCA |
|  | GTTTCTTGATCCATCATCTTCCTG |
| *AGL40* | AATATACTCACTGAGGTGCTG |
|  | ACCAGTTTCCTACTTGTTCTC |
| *AGL28* | GAGTCTTTAACTGAGGTGATGG |
|  | GCTAAGTTGAGTTCTGTTGGAG |
| *MEA* | GGTGAGGCACTAGAATTGAGCAGT |
|  | CCATAGTCCTGCCCAACCG |
| *FIS2* | CCATAGTCCTGCCCAACCG |
|  | CCATAGTCCTGCCCAACCG |
| *MEO* | GTGGCTCGTAAGATGCAGATG |
|  | AGCGAGGATCTTTCCAGTGAC |
| *PP2A* | TAACGTGGCCAAAATGATGC |
|  | GTTCTCCACAACCGCTTGGT |
| *ACT11* | GGAACAGTGTGACTCACACCATC |
|  | AAGCTGTTCTTTCCCTCTACGC |

Primers for quantification of genomic *agl62* alleles

| mutant specific | ATTTTGCCGATTTCGGAAC |
| --- | --- |
|  | CGAGTTGAGATAACGCAAGTTCC |
| wild-type specific | ATGCCGATGAAATGTTATAGAATC |
|  | CTTTAACTCGTCGTACTTCTTT |
| mutant/wild-type unspecific | AACAGAGAAAAAGAAGTACGACGAG |
|  | CGAGTTGAGATAACGCAAGTTCC |

Primers for genotyping

| *osd1* (WT specific) | CGTCACTCTCCCCAAGAAAG |
| --- | --- |
|  | GGCTAAGCAAGCCTGCTAT |
| *osd1* (mutant specific) | GGCTAAGCAAGCCTGCTAT |
|  | TCCGTTCCGTTTTCGTTTTTTAC |
| *mea* (WT specific) | GCTGATGGCTGAAAGTGATTC |
|  | GTCTTAAGCCCCCGAAGTATG |
| *mea* (mutant specific) | GCCTTTTCAGAAATGGATAAATAGCCTTGCTTCC |
|  | GTCTTAAGCCCCCGAAGTATG |
| *fis2* (WT specific) | TGTTGTTTCCATGATTTCTTTTTC |
|  | AAACCGAACCAGTTTTCATACC |
| *fis2* (mutant specific) | AAACCGAACCAGTTTTCATACC |
|  | ATTTTGCCGATTTCGGAAC |
| *agl62* (WT specific) | TGAGCTTTGCACACTTTGTGGTG |
|  | AAGCATTGTTTCCAAAGGGTGG |
| *agl62* (mutant specific) | AAGCATTGTTTCCAAAGGGTGG |
|  | ATTTTGCCGATTTCGGAAC |
